# Priority actions for Fusarium head blight resistance in durum wheat: Insights from the wheat initiative

**DOI:** 10.1002/tpg2.20539

**Published:** 2025-01-06

**Authors:** Ambra Viviani, Jemanesh K. Haile, W. G. Dilantha Fernando, Carla Ceoloni, Ljiljana Kuzmanović, Dhondup Lhamo, Yong‐Qiang Gu, Steven S. Xu, Xiwen Cai, Hermann Buerstmayr, Elias M. Elias, Alessia Confortini, Matteo Bozzoli, Gurcharn Singh Brar, Yuefeng Ruan, Samia Berraies, Walid Hamada, Safa Oufensou, Malini Jayawardana, Sean Walkowiak, Salim Bourras, Monika Dayarathne, Julio Isidro y Sánchez, Fiona Doohan, Agata Gadaleta, Ilaria Marcotuli, Xinyao He, Pawan K. Singh, Susanne Dreisigacker, Karim Ammar, Valentyna Klymiuk, Curtis J. Pozniak, Roberto Tuberosa, Marco Maccaferri, Barbara Steiner, Anna Maria Mastrangelo, Luigi Cattivelli

**Affiliations:** ^1^ Department of Agricultural Sciences University of Bologna Bologna Italy; ^2^ Crop Development Centre and Department of Plant Sciences University of Saskatchewan Saskatoon Saskatchewan Canada; ^3^ Department of Plant Science University of Manitoba Winnipeg Manitoba Canada; ^4^ Department of Agriculture and Forest Sciences (DAFNE) University of Tuscia Viterbo Italy; ^5^ USDA‐ARS, Crop Improvement and Genetics Research Unit, Western Regional Research Center Albany California USA; ^6^ USDA‐ARS, Wheat, Sorghum & Forage Research Unit Lincoln Nebraska USA; ^7^ Department of Agronomy and Horticulture University of Nebraska Lincoln Nebraska USA; ^8^ Department of Agrobiotechnology Tulln University of Natural Resources and Life Sciences Vienna Tulln Austria; ^9^ Department of Plant Sciences North Dakota State University North Dakota Fargo USA; ^10^ Ag, Food & Nutr Science Department University of British Columbia (UBC) Vancouver British Columbia Canada; ^11^ Swift Current Research and Development Center, Agriculture and Agri‐Food Canada Swift Current Saskatchewan Canada; ^12^ Institut National Agronomique de Tunisie 43 Tunis Tunisia; ^13^ Desertification Research Center (NRD) Università degli Studi di Sassari Sassari Italy; ^14^ Canadian Grain Commission Winnipeg Manitoba Canada; ^15^ Department of Plant Biology Swedish University of Agricultural Sciences Uppsala Sweden; ^16^ Centro de Biotecnología y Genómica de Plantas Universidad Politécnica de Madrid Madrid Spain; ^17^ School of Biology and Environmental Science UCD Earth Institute and UCD Institute for Food and Health, Belfield Dublin Ireland; ^18^ Department of Soil, Plant and Food Sciences University of Bari Aldo Moro Bari Italy; ^19^ International Maize and Wheat Improvement Center (CIMMYT) Texcoco Edo de Mexico Mexico; ^20^ CREA ‐ Research Centre for Cereal and Industrial Crops Foggia Italy; ^21^ CREA ‐ Research Centre for Genomics and Bioinformatics Fiorenzuola d'Arda (PC) Italy

## Abstract

Fusarium head blight (FHB), mainly caused by *Fusarium graminearum* and *Fusarium culmorum*, is a major wheat disease. Significant efforts have been made to improve resistance to FHB in bread wheat (*Triticum aestivum*), but more work is needed for durum wheat (*Triticum turgidum* spp. *durum*). Bread wheat has ample genetic variation for resistance breeding, which can be readily exploited, while durum wheat is characterized by higher disease susceptibility and fewer valuable resistance sources. The Wheat Initiative – Expert Working Group on Durum Wheat Genomics and Breeding has promoted a scientific discussion to define the key actions that should be prioritized for achieving resistance in durum wheat comparable to that found in bread wheat. Here, a detailed state of the art and novel tools to improve FHB resistance in durum are presented, together with a perspective on the next steps forward. A meta‐analysis grouping all quantitative trait loci (QTL) associated with FHB resistance in both bread and durum wheat has been conducted to identify hotspot regions that do not overlap with *Rht* alleles, which are known to negatively correlate with FHB resistance. A detailed list of QTL related to FHB resistance and deoxynivalenol contamination and durum lines carrying different sources of FHB resistance are provided as a strategic resource. QTL, closely linked markers and durum wheat lines carrying the useful alleles, can be selected to design an effective breeding program. Finally, we highlight the priority actions that should be implemented to achieve satisfactory resistance to FHB in durum wheat.

## Core Ideas

- Durum wheat has higher susceptibility and fewer resistance sources to Fusarium head blight (FHB) than bread wheat.
- A continued investment is needed in reproducible phenotypic screening of germplasm for FHB resistance traits.
- Improved lines generated in various programs around the globe should be exchanged.
- The quantitative trait loci (QTL) meta‐analysis presents the most complete and updated list of all QTLs governing reaction to FHB in wheat.
- A coordinated pathogen and toxin surveillance is encouraged to monitor the evolution of *Fusarium* populations.

**Abbreviations**
15‐ADON: 15‐acetyl‐deoxynivalenol
3‐ADON: 3‐acetyl‐deoxynivalenol
4‐ANIV: 4‐acetylnivalenol
CI: confidence interval
CIMMYT: International Maize and Wheat Improvement Center
CYP450s: cytochrome P450s
DAS: diacetoxyscirpenol
DELLA: aspartic acid–glutamic acid–leucine–leucine–alanine
DMI: demethylation inhibitor
DON: deoxynivalenol
FCR: Fusarium crown rot
FFSC: *F. fujikuroi* species complex
FHB: Fusarium head blight
Fhb1‐In1: FHB inhibitor‐1
FRG: *F. graminearum*‐responsive gene
FSAMSC: *Fusarium sambucinum* species complex
FTSC: *F. tricinctum* species complex
GA: gibberellic acid
GS: genomic selection
GSH: glutathione
GST: glutathione‐S‐transferase
GWAS: genome‐wide association studies
KASP: kompetitive allele specific PCR
MAP: mitogen‐activated protein
MAS: monoacetoxyscirpenol
MRP: multidrug resistance protein
NAC: no apical meristem (NAM)
NEO: neosolaniol
NIV: nivalenol
OSP24: orphan secreted protein 24
Ph1: pairing homeologous 1
PME1: pectin methylesterase enzyme 1
QoI: quinone outside inhibitors
QTL: quantitative trait loci
Rht‐1: reduced height‐1
SNP: single nucleotide polymorphism
SnRK1α: non‐fermenting‐1 (SNF1)‐related protein kinase 1 catalytic subunit α
TaFROG: *Triticum aestivum* Fusarium Resistance Orphan Gene
TaNACL‐D1: *Triticum aestivum* NAC‐like D1
UGTs: UPD‐glycosyl transferases
ZEA: zearalenone

## 1 INTRODUCTION

Durum wheat (*Triticum turgidum* L. ssp. *durum* (Desf.) Husnot) faces abiotic and biotic stresses, which limit grain yield and grain quality. Durum, as well as bread wheat, is affected by many fungal pathogens, such as the cereal rusts, powdery mildew, tan spot, *Septoria tritici* blotch, *Septoria nodorum* blotch, *Fusarium* species, and more. *Fusarium* species may infect the crown (causing Fusarium crown rot [FCR]) as well as the head (Fusarium head blight [FHB]) of wheat plants. FCR, mainly caused by *Fusarium pseudograminearum* and *Fusarium culmorum*, is a disease occurring in dry (rainfed) growing regions characterized by visible darkening of the basal parts of the plant and bleaching of wheat stems and spikes, due to the termination of sap and nutrient supply. Depending on the timing of infection, white heads may contain few, small, or no seeds at all with significant yield losses, but without major concern for grain toxin contamination (Covarelli et al., 2015; Fan et al., 2021).

FHB, mainly caused by *Fusarium graminearum* and, to same extent, by *F. culmorum* and additional fungal species (Table 1), is one of the most devastating wheat diseases. While substantial research and breeding efforts have been dedicated to better understand and mitigate FHB in bread wheat (Buerstmayr et al., 2020), it remains more challenging in durum wheat. In bread wheat, ample genetic variation has been described and can readily be exploited for resistance breeding, while durum wheat has a higher disease susceptibility, and the sources of resistance are limited (Haile et al., 2019; Prat et al., 2014). Savary et al. (2019) estimated a global yield loss of 21.5% in wheat due to FHB incidences with particularly high losses in China, US Midwest, Canada, and South America. Besides yield losses, FHB pathogens contaminate the grain with a range of toxic fungal metabolites known as mycotoxins that pose a health risk to humans and animals (D'Mello et al., 1997).

**TABLE 1:** *Fusarium* species associated with Fusarium head blight (FHB) in durum wheat and corresponding mycotoxins.

*Fusarium* species	DON	NIV	4ANIV	T2	HT2	NX2	NX3	DAS	MAS	ZEA	NEO	Enniatin	Moniliformin	Beauvericin	Aurofusarin	Siccanol	Butenolide	Fusarenone	Fusaric acid	Fusaproliferin	Fuminosin	References
*F. acuminatum*																						Alisaac and Mahlein (2023); Fakhfakh et al. (2011a); Haidukowski et al. (2022)
*F. avenaceum*																						Tittlemier et al. (2013); Visconti and Pascale (2010)
*F. cerealis*																						Palacios et al. (2021)
*F. culmorum*																						Covarelli et al. (2015); Visconti and Pascale (2010)
*F. equiseti*																						Alisaac and Mahlein (2023); Alkadri et al. (2013); Covarelli et al. (2015)
*F. graminearum*																						Alisaac and Mahlein (2023); Bamforth et al. (2022); Visconti and Pascale (2010)
*F. langsethiae*																						Somma et al. (2022); Visconti and Pascale (2010)
*F. poae*																						Alisaac and Mahlein (2023); Covarelli et al. (2015)
*F. proliferatum*																						Alisaac and Mahlein (2023); Gorczyca et al. (2018)
*F. pseudograminearum*																						Chakroun et al. (2022)
*F. sambucinum*																						Alisaac and Mahlein (2023); Visconti and Pascale (2010)
*F. sporotrichioides*																						Alisaac and Mahlein (2023); Somma et al. (2022); Visconti and Pascale (2010)
*F. subglutinans*																						Alisaac and Mahlein (2023); Gorczyca et al. (2018); Jestoi (2008)
*F. tricinctum*																						Alisaac and Mahlein (2023); Beccari et al. (2018); Senatore et al. (2021)
*F. verticillioides*																						Alisaac and Mahlein (2023); Ferrigo et al. (2016); Krnjaja et al. (2022); Mylona et al. (2019)

*Note*: The blue color indicates the presence of the corresponding toxin.

Abbreviations: 4‐ANIV, 4‐acetyl‐nivalenol; DAS, diacetoxyscirpenol; DON, deoxynivalenol; MAS, monoacetoxyscirpenol; NEO, neosolaniol; NIV, nivalenol; zearalenone (ZEA).

Although post‐harvesting cleaning methodologies can be used to remove the fusarium damaged kernels and indirectly reduce the mycotoxin concentration in the fraction used for human consumption, the problem is far from being solved and, currently, there is no “silver bullet” solution for durum wheat (Cheli et al., 2013). An integrated FHB management including good agronomic practices, fungicide applications, and cultivation of less susceptible cultivars, is the most cost‐effective and environmentally friendly option. However, durum wheat overall is more susceptible than bread wheat, and resistant cultivars are lacking. With the expansion of durum wheat cultivation beyond the traditional summer‐dry Mediterranean climate to more humid and cooler climates that are more favorable for *Fusarium* spp., the Fusarium disease pressure is consequently also increasing. In high disease pressure years and regions, heavy epidemics in durum wheat can result in severe mycotoxin contamination, exciding legislative limits. The Canadian Grain Commission's survey found significant variation on the occurrence and amount of FHB and associated toxins by the *Fusarium* spp. infestation with estimated economic losses due to FHB predicted to be 1 billion CAD in 2016 (Chin et al., 2023; Dawson, 2016).

The Wheat Initiative – Expert Working Group on Durum Wheat Genomics and Breeding met in Bari (Italy) in October 2023 to discuss current knowledge about FHB in durum wheat and outlined the actions that should be prioritized to achieve improved (higher) resistance levels comparable to bread wheat. A coordinated pathogen and toxin surveillance, an international ring trial for a comparative evaluation of available sources of resistance across a range of different environments and an extensive genomic‐assisted breeding design to capture major and minor quantitative trait loci (QTL) have been highlighted as the current limiting factors to support progress in FHB‐resistant breeding in durum wheat. This study complements recently published reviews on FHB resistance in wheat (Buerstmayr et al., 2020; Steiner et al., 2017) and specifically durum wheat (Haile et al., 2019; Prat et al., 2014) and suggests avenues for future research.

**Core Ideas**

- Durum wheat has higher susceptibility and fewer resistance sources to Fusarium head blight (FHB) than bread wheat.
- A continued investment is needed in reproducible phenotypic screening of germplasm for FHB resistance traits.
- Improved lines generated in various programs around the globe should be exchanged.
- The quantitative trait loci (QTL) meta‐analysis presents the most complete and updated list of all QTLs governing reaction to FHB in wheat.
- A coordinated pathogen and toxin surveillance is encouraged to monitor the evolution of *Fusarium* populations.

## 2 THE FHB SPECIES COMPLEX

FHB is caused by a complex of up to 17 different fungal species (Karlsson et al., 2021). While many taxa associated with FHB belong to the genus *Fusarium*, the complex also includes species from the genus *Microdochium*, such as *Microdochium majus* and *Microdochium nivale* (McCormick et al., 2013). The FHB species isolated from durum wheat vary across environments, including location and year, likely due to many interacting factors required for disease development, such as climate/weather, host genetics, and inoculum (Bamforth et al., 2022; Covarelli et al., 2015; Haidukowski et al., 2022; Table 1).

*Fusarium* species are ubiquitous endophytic colonizers of cereals and grasses, found in all wheat tissues, from roots to seeds. Comparative genomic studies support the hypothesis that pathogenic *Fusarium* taxa evolved from endophytic *Fusarium* species (Hill et al., 2022; L.‐J. Ma et al., 2010). It has also been shown that *Fusarium* genomes show no clear signatures of lifestyle transitions, indicating a high degree of lifestyle plasticity (Hill et al., 2022). *Fusarium* species exhibit a hemi‐biotrophic behavior during infection, characterized by an initial biotrophic phase followed by a necrotrophic phase. Since the pathogens initially infect the floral tissues, the critical stage of FHB infection in wheat occurs from anthesis to soft dough stage (Alisaac & Mahlein, 2023; Haile et al., 2019; M. McMullen et al., 2012), with optimal environmental conditions characterized by 16°C–30°C and at least 10 h/day of high humidity. FHB can cause several symptoms in the kernels, including lesions, spike discoloration (Alisaac & Mahlein, 2023), kernel abortion, and shriveling with white or pink discoloration. These symptoms highlight the disease's destructive potential and its capacity to dominate other microbes in the flowers and kernels. This dominance is very important from an epidemiological perspective because wheat is affected by a variety of diseases at earlier developmental stages which can affect secondary infections by FHB. Studies have shown that primary infection by the wheat pathogen *Zymoseptoria tritici* induces systemic susceptibility, leading to the breakdown of the host immune system, and allowing for secondary infection with a non‐adapted pathogen for which wheat is normally a non‐host (Seybold et al., 2020). Furthermore, such response was shown to transcend tissue and organ barriers through systemic reprogramming of the wheat metabolome and the endophytic microbiome in tissues that were not even infected with *Z. tritici* (Seybold et al., 2020). This example highlights another gap in our understanding of the plant immune response to FHB based on systemic signals that can be triggered locally to propagate across tissues and organs from roots to shoots (Ko & Helariutta, 2017; Vlot et al., 2021). Thus, considering that FHB infections are initiated late in the season, these are likely to occur on primed hosts where *Fusarium* must “make space” by counteracting plant immune responses triggered by other microbes (not only pathogens) locally in the anthers, or systemically by pathogens and microbes colonizing other tissues in the shoot or the roots. In fact, it was shown that FHB infections induce significant changes on the endophytic spike communities in wheat, thus providing direct evidence for the evolution of microbiome competition traits in the FHB complex. This underscores the importance of recognizing the microbiome interaction component in basic epidemiology and FHB resistance, irrespective of the level of host immunity (Rojas et al., 2020).

A major problem associated with FHB are mycotoxins, toxic secondary metabolites, harmful to both humans and animals whose accumulation generally correlates with FHB severity (Scarpino & Blandino, 2021; M. X. Zhao et al., 2018). Evidence suggests that *Fusarium* mycotoxins act as antimicrobial compounds against other microbes and could possibly represent a reminiscence of wild grass ecosystems, where mycotoxin accumulation provided protection against herbivores (Sweany et al., 2022).

Trichothecenes are one of the major classes of mycotoxins produced by *Fusarium* spp. Two types of trichothecenes are linked to FHB in durum wheat: type A trichothecenes, like T2 or HT2, and type B trichothecenes such as deoxynivalenol (DON) and nivalenol (NIV; Covarelli et al., 2015; McCormick et al., 2013). Recently, NX‐2, a novel type A trichothecene, has been found in wild grasses, bread, and durum wheat (Crippin et al., 2019; Foroud et al., 2019; A. Kelly et al., 2016). Several *Fusarium* species, including *Fusarium langsethiae, Fusarium poae*, and *Fusarium sporotrichiodes*, are responsible for producing type A trichothecenes, and among these species, *Fusarium langsethiae* and *Fusarium sporotrichioides* are the primary producers of T‐2 and HT‐2 toxins in durum wheat (Haidukowski et al., 2022; Isidro‐Sánchez et al., 2020; Somma et al., 2022).

The majority of*. F. graminearum* strains produce type B trichothecenes, including DON, 15‐acetyl‐DON (15‐ADON), 3‐acetyl‐DON (3‐ADON), and NIV (A. Kelly et al., 2016; Varga et al., 2015), with DON being the predominant mycotoxin in durum wheat (Bamforth et al., 2022; Bryła et al., 2018). Other major fungal species responsible for DON contamination are *F. culmorum* and *F. cerealis* (Desjardins & Proctor, 2007). The presence of NIV in grains of durum wheat cultivars was also described (Gorczyca et al., 2018).

Zearalenone, a non‐steroidal estrogenic mycotoxin, is another toxin found in durum wheat (Zaied et al., 2012), produced by specific strains of *F. graminearum* and *F. culmorum* (Bennett & Klich, 2003). A survey of Tunisian durum wheat reported by Zaied et al. (2012) showed 23% of the samples exceeded the mycotoxin limit imposed by EU legislation, suggesting that monitoring zearalenone content in wheat grain and wheat products is as important as monitoring DON. Finally, moniliformin, produced by *Fusarium avenaceum* and *Fusarium acuminatum*, has been detected in several studies on durum wheat, conducted in Austria (Adler et al., 1995) and Canada (Tittlemier et al., 2013).

Although mycotoxins actively accumulated in wheat kernels during early phases, their level decreases during food processing steps, from raw and uncleaned durum wheat grain to processed food (Haile et al., 2019; Visconti & Pascale, 2010; Visconti et al., 2004). However, toxins are not completely eliminated from the final food product, and many countries, including the United States and the European Union, implement continuous monitoring, guidelines, and legal limits to ensure food safety (Visconti et al., 2004).

## 3 A SHORT GLIMPSE INTO HISTORY OF FHB

Severity reactions of wheat to FHB were first reported by Arthur (1891). He observed that differences in flowering time and plant vigor were associated with FHB severity and that the infection started from the wheat florets. An early comprehensive report about FHB in wheat and other cereals dates back to the beginning of the 20th century (Atanasoff, 1920). These manuscripts described FHB symptoms and infection through the florets, and their conclusions remain valid today. Atanasoff (1920) also reported differences in resistance among bread wheat genotypes and observed that durum wheat was more susceptible than bread wheat.

During the first half of the last century, many varieties, breeding lines, and germplasm accessions were evaluated for FHB resistance (Schroeder & Christensen, 1963, and references therein). For example, Christensen et al. (1929) evaluated 350 spring wheat genotypes in Minnesota (USA), using an artificial inoculation protocol by spraying fungal spore suspensions at flowering time and assessed the percentages of affected heads and infected seeds after harvest. They reported significant phenotypic variation of FHB infection with the durum cv. Mindum, a selection developed at the Minnesota Agricultural Experiment Station in 1917 (Clark & Martin, 1923) being the most resistant, averaging 25% infected heads over six testing seasons. However, this value was rather low compared to bread wheat varieties. The susceptible bread wheat cv. Kitchener (developed by Agriculture Canada in 1911) had a similar percentage infection (26.7%), while the resistant bread wheat Glyndon Five only had 4% infected spikes on average. Christensen et al. (1929) summarized their observations on durum wheat as follows: “The durum wheats as a class were decidedly more susceptible than the common wheats. In 1927 the percentage of infection on different varieties of durum ranged from 1 to 100, although most of the more than 200 varieties tested from 1925 to 1928 were susceptible.”

This historical report pinpoints the main problem: durum wheat lacks useful sources of FHB resistance. Since then, evaluations of cultivars, breeding lines, and genetic resources have not changed this picture until the early 2000s, with most durum wheat cultivars appearing moderately to highly susceptible (Clarke et al., 2010; Miedaner & Longin, 2014). Even large efforts to identify durum wheat accessions with enhanced resistance have shown limited success. Elias et al. (2005) screened large collections including several thousand of durum wheat accessions but failed to identify resistant lines. A later evaluation of accessions from the International Maize and Wheat Improvement Center (CIMMYT) and and International Center for Agricultural Research in the Dry Areas (ICARDA) identified only five Tunisian durum wheat landraces with moderate resistance (Elias et al., 2005; Huhn et al., 2012), while only four accessions were found to be promising candidates for FHB resistance breeding in a screening of Syrian landraces after spray inoculation (Talas et al., 2011).

The scarcity of truly FHB‐resistant sources in durum wheat has not yet been clearly elucidated. One possible explanation is that the cultivated durum germplasm, mostly descending from landraces native to the hot and summer‐dry Mediterranean basin, has not been exposed to relevant disease pressure, and therefore resistance mechanisms did not evolve (Kuzmanović et al., 2019). Furthermore, it has been suggested that the expression of FHB resistance in durum wheat is compromised by the presence of susceptibility factors and/or suppressor genes in its genome (Garvin et al., 2009; Ghavami et al., 2011).

## 4 SOURCES OF RESISTANCE

### 4.1 Major QTL/genes for FHB resistance

Due to the hemi‐biotrophic behavior of *F. graminearum* and *F. culmorum*, along with the complex and diverse components (referred to as resistance types) of wheat FHB disease response, FHB resistance is a typical quantitative and complex trait (Buerstmayr et al., 2019; Mesterhazy, 2020). Wheat FHB response includes multiple components: type‐I resistance to initial infection through open florets and anthers, type‐II resistance to pathogen spread within the spike, DON mycotoxin accumulation in kernels, response to DON, and fusarium damaged kernels (reviewed in Mesterhazy, 2020). The quantitative inheritance of FHB resistance affects the response to selection, and how genomic tools can be leveraged and deployed in combination with phenotypic selection to accelerate breeding for resistance (Buerstmayr et al., 2020; Mesterhazy, 2020).

Even though FHB response is a typical quantitative trait, the heritability (*H* ^2^ broad sense) of multiple FHB traits across environments ranged from 0.23 to 0.92 with an average of 0.67 (±0.20) and a median of 0.76 (Supporting Information 1) in a survey of 13 studies targeting tetraploid wheat germplasm. Medium‐to‐high *H* ^2^ values can be obtained when phenotyping is conducted using appropriate experimental methodologies based on repeated artificial inoculations with selected isolates, mist irrigation, and repeated scoring of the *Fusarium* incidence and severity to generate robust and integrative area under the disease progress curve values. So far, nine major loci and many QTL with minor effects (Venske et al., 2019) have been reported in the wheat gene pool, six from *Triticum* accessions and three from wild relatives (Table 2).

**TABLE 2:** Major loci for resistance to Fusarium head blight (FHB) reported in the wheat gene pool.

Major loci for FHB resistance	Chromosome	Origin	Reference	Note
*Fhb1 (QFhs.ndsu3BS)*	3BS	Type‐II resistance from bread wheat cv. Sumai‐3	Anderson et al. (2001); Buerstmayr et al. (2002, 2003); Cuthbert et al. (2006); Waldron et al. (1999)	*Fhb1* had a strong effect on resistance. As an example, it explained 41.6% and 24.8% of the resistance to FHB in the Sumai 3/Stoa and ND2603/Butte 86 populations, respectively (Anderson et al., 2001).
*Fhb2*	6BS	Type‐II resistance from bread wheat cv. Sumai‐3	Anderson et al. (2001); Cuthbert et al. (2007)	*Fhb2* showed a limited effect on resistance. As an example, it explained 9.2% and 4.9% of the resistance to FHB in the Sumai 3/Stoa and ND2603/Butte 86 populations, respectively (Anderson et al., 2001).
*Fhb3*	4BL	*Leymus racemosus*	Qi et al. (2008)	The FHB resistance conferred by translocation T7AL·7Lr#1S in the CS background was reported similar to that of Sumai 3 by Qi et al. (2008).
*Fhb4*	7AL	Type‐I resistance from bread wheat cv. Wangshuibai	S. Xue et al. (2010)	In a BC3F2 population derived from the cross of a Qfhi.nau‐4B near isogenic line (NIL) with susceptible cultivar Mianyang 99–323, the most resistant class had over 60% less infection than the susceptible one (S. Xue et al., 2010).
*Fhb5* (*Qfhb.rwg*, most likely synonym to *Qfhs.ifa‐5A*)	5A	Type‐I resistance from bread wheat cvs. Sumai‐3 and Wangshuibai	Buerstmayr et al. (2003, 2018); Steiner, Buerstmayr, et al. (2019); S. Xue et al. (2011)	Introgression of *Fhb5* into durum wheat has frequently been associated with increased sterility (Hermann Buerstmayr, unpublished results).
*Fhb6*	1AS	Type‐II resistance from *Elymus tsukushiensis*	Cainong et al. (2015)	Plant progenies homozygous for *Fhb6* had a disease severity rating of 7% compared to 35% for the null progenies (Cainong et al., 2015).
*Fhb7*	7AL, 7BL, 7DL	*Thinopyrum ponticum*/*Th. Elongatum*	Forte et al. (2014); J. Guo et al. (2015); Kuzmanović et al. (2019); H. Wang et al. (2020); X. Zhang et al. (2011); W. Zhang, Danilova, et al. (2022)	Infection outcomes confirmed previous observations in bread wheat, with >90% reduction of disease severity associated with *Fhb‐7EL* (Kuzmanović et al., 2019).
*Fhb8*	7D	Bread wheat cv. Wangshuibai	X. Wang et al. (2024)	Resistance evaluations in recombinant inbred lines carrying *Fhb8* on 7D have mapped the locus in a 1.0‐cM *Xwgrb1500*‐*Xwgrb1559* interval (from 93.9–96.5 Mb in CS) in co‐segregation with marker *Xwgrb1587* (X. Wang et al., 2024).
*Fhb9*	2DL	Type‐II resistance from bread wheat line Ji5265	F. Zhang et al. (2024)	*Fhb9* explained 26%–30% of the phenotypic variation in a recombinant inbred line population derived from Shi4185×Shijiazhuang 8 (F. Zhang et al., 2024).

The gene controlling *Fhb1*‐mediated resistance to fungal spreading encodes a putative nuclear‐localized, histidine‐rich calcium‐binding protein (G. Q. Li et al., 2019; Su et al., 2019). The wild‐type allele is characterized by a 786‐bp open reading frame, while the allele associated with type‐II FHB resistance in Sumai‐3 carries a 752‐bp deletion that encompasses the 5′‐flanking region and the 5′‐end of the coding sequence (G. Q. Li et al., 2019; Su et al., 2019).

Specific wheatgrass chromosomes contributing substantial FHB resistance have been introgressed in wheat by means of addition or substitution lines carrying single alien chromosomes in a wheat background. The 7Lr ditelosomic line carrying a *Leymus racemosus* region orthologous to chromosome 7A showed FHB resistance due to the *Fhb3* gene (Qi et al., 2008; L. S. Wang & Chen, 2008). A short segment of *Elymus tsukushiensis* harboring the *Fhb6* gene was stably incorporated into the distal part of bread wheat chromosome 1AS (Cainong et al., 2015). Other sources of resistance were found in wheat–wheatgrass introgression lines carrying genomic regions from *Leymus multicaulis*, *Elymus rectisetus*, and *Elymus repens* (Dou et al., 2012; McArthur et al., 2012; Zeng et al., 2013; X. Zhang et al., 2010).

The *Thinopyrum* genus is the most exploited alien germplasm in wheat breeding and includes several sources of FHB resistance. This includes the addition of single *Thinopyrum junceum* chromosomes from homoeologous groups 2 and 5 to the bread wheat genome (McArthur et al., 2012), as well as the incorporation of a pair of *Thinopyrum elongatum* group 1 chromosomes (1E) into the durum wheat cv. Langdon (Jauhar et al., 2009; Jauhar & Peterson, 2011). Potentially highly efficient QTL for FHB resistance appear those originating from the *el_2_* accession of decaploid *Th. ponticum* (Shen & Ohm, 2007) and from the diploid *Th. elongatum* (Oliver et al., 2005; Shen et al., 2004). Genetic mapping along their homoeologous group 7 long arms, 7el_2_L and 7EL, respectively (Ceoloni et al., 2017; Forte et al., 2014; J. Guo et al., 2015; W. Zhang, Danilova, et al., 2022), and their partial sequence and functional homology (X. Guo et al., 2022; Konkin et al., 2022; H. Wang et al., 2020; W. Zhang, Danilova, et al., 2022), indicate that the *Fhb7el_2_* and *Fhb7E* loci (sometimes collectively referred to as *Fhb7*) are likely orthologs. When transferred via chromosome engineering on the 7AL arm of both bread and durum wheat, the two loci showed a comparable and remarkable efficiency in decreasing the severity of FHB disease by >80%, that is, the number of diseased florets following *F. graminearum* infection (Ceoloni et al., 2017; Forte et al., 2014; Kuzmanović et al., 2019). Furthermore, an *Fhb7* allele from *Th. elongatum* chromosome 7EL was transferred also to the B genome of bread wheat (W. Zhang, Danilova, et al., 2022) providing an additional source for the introgression of *Fhb7* in durum wheat.

The *Fhb7*‐resistant phenotypes also show decreased fungal biomass accumulation, normally developed grains and extremely low DON content (>800‐fold decrease vs. susceptible plants in durum wheat; Kuzmanović et al., 2019) due to an early block of disease spread beyond the inoculation point resulting in a near immunity. A finding that could be game changer in FHB resistance breeding, though deployment in applied breeding is yet pending.

The *Fhb7* alleles within the small alien chromosomal segments generally exhibit a monogenic‐like inheritance pattern when integrated into the wheat genome through translocations because they normally do not recombine with their homoeologous counterparts of wheat in the presence of *Ph1*. This boosts the utility of *Fhb7* in wheat breeding, especially in durum wheat with complex epistatic effects with FHB resistance QTL (Zhu et al., 2022).

Glutathione (GSH) biosynthesis and metabolism are among the major mechanisms determining *Fhb7E* resistance (Fanelli et al., 2023), particularly through DON conjugation with glutathione (DON‐GSH). This supports earlier evidence of horizontal transfer of a glutathione‐S‐transferase (*GST*) gene from an unrelated endophytic fungus of the *Epichloë* genus, into the *Fhb7* locus of *Thinopyrum* species (H. Wang et al., 2020). The GST function involves the formation of a de‐epoxidated DON–GSH adduct, which irreversibly impairs DON‐toxicity (Uhlig et al., 2016; Yang et al., 2024; L. Zhao et al., 2024) and hence decreases pathogen virulence.

Beside loci coding for FHB resistance, morphological traits, such as plant height and anther extrusion, have frequently been associated with FHB severity. Taller plants are less susceptible than short ones (Kirana et al., 2023; Prat et al., 2017), and genotypes with a higher degree of anther extrusion are generally less susceptible than those with a high degree of retained anthers in bread wheat (Buerstmayr & Buerstmayr, 2015; Lu et al., 2013; Skinnes et al., 2008; Steiner, Buerstmayr, et al., 2019). It has been reported that semi‐dwarf genes such as *Rht‐B1b* and *Rht‐D1b* are also linked to reduced anther extrusion in bread wheat (X. He et al., 2016), making the selection of modern semi‐dwarf genotypes with high anther extrusion more challenging. This association may be explained at physiological level considering that gibberellic acid (GA) has been shown to stimulate the elongation of anther filament in *Arabidopsis*, while DELLA proteins, which are orthologous to wheat *Rht‐1* gene products, repress it (Cheng et al., 2004). Therefore, it can be argued that the GA insensitive mutants *Rht‐B1b* and *Rht‐D1b* have a similar function in wheat. In light of this finding, *Rht‐B1b* and *Rht‐D1b* could exert pleiotropic effects, leading to low anther extrusion, which in turn increases type‐I FHB susceptibility (X. He et al., 2016).

While plant height cannot be increased beyond an agronomically acceptable level due to an increased lodging, anther extrusion appears a good selection target for indirect selection toward increased FHB resistance, and variation for this trait is available in the durum wheat gene pool (Akel et al., 2019).

### 4.2 Minor loci/genes associated with FHB resistance and susceptibility

A range of additional minor loci/genes have been found to enhance FHB resistance in wheat. Some of these genes have been directly associated with DON tolerance, while others have yet to be proven to directly affect it (reviewed in Perochon & Doohan, 2024; Supporting Information 2). Characterizing counterparts of DON responsive and/or tolerance genes in durum wheat, or introgressing these genes from hexaploid wheat, offers new breeding strategies for the enhancement of FHB resistance in durum wheat. DON and other trichothecenes are phytotoxic compounds that promote plant cell death, facilitating the necrotrophic phase of FHB where the pathogen spreads within host tissue (Audenaert et al., 2014; Gunupuru et al., 2017). As phytotoxins, trichothecenes activate host defenses (Gunupuru et al., 2017), and cereal genes of diverse function have been shown to reduce their phytotoxic effects, contributing to the control of FHB disease.

DON tolerance mechanisms include detoxification genes; transcription factors; and novel, evolutionary divergent genes. Classic detoxification genes, such as UPD‐glycosyl transferases (UGTs), *GSTs*, cytochrome P450s (CYP450s), and multidrug resistance proteins (MRPs), have been associated with and shown to enhance FHB resistance. Wheat genotypes have the potential to convert DON to the less phytotoxic DON‐3‐glucoside (Cirlini et al., 2013), a reaction catalyzed by UGTs (Poppenberger et al., 2003), although the conversion rate of DON to DON‐3‐glucose was higher in hexaploid than in durum wheat lines carrying *Fhb1* and *Fhb7* QTL (Lemmens et al., 2005; Kluger et al., 2015; H. Wang et al., 2020).

In addition to detoxification mechanisms, the suppression of toxin production may be a useful strategy for controlling FHB. Benzoxazinoid phytoalexins are produced by cereals and have demonstrated DON suppression activity in vitro (Etzerodt et al., 2015), which likely also operates *in planta*. Detoxification of benzoxazolinones aids *Fusarium* overcome wheat defenses (Baldwin et al., 2019).

Beyond the classic detoxification genes, genes involved in lipid transfer, stress signaling, transcription factors, and evolutionary divergent novel genes have also been proven to help wheat overcome the effects of DON and thus enhance FHB resistance. The central stress regulator *TaSnRK1α*/*TaSnRK1a* and a novel *Poaceae*‐divergent NAC‐like transcription factor TaNACL‐D1 both interact with an evolutionary divergent *Pooideae‐*specific protein, TaFROG, and all three contribute to DON tolerance in wheat (Perochon et al., 2015, Perochon, Kahla, et al., 2019; Perochon, Váry, et al., 2019). Given its evolutionary divergence, this is an interesting complex for further study. *TaFROG* resides within a genomic hotspot adjacent to the *Poaceae*‐specific *RZ53* that is highly co‐expressed with *TaFROG* (Perochon et al., 2021). TaFROG was shown to compete with the *Fusarium* orphan protein effector Osp24 to stabilize TaSnRK1α (Jiang et al., 2020). This reinforces the importance of mycotoxins within the co‐evolution of *Fusarium* and cereals. From an FHB resistance breeding perspective, these studies collectively demonstrate the importance of a DON‐tolerance gene in counteracting the effects of a fungal effector and thus reduce disease severity.

One of the hypotheses about tetraploid wheat vulnerability is that it may carry some susceptibility factors and/or resistance gene suppressors. A susceptibility gene factor was identified by Giancaspro et al. (2016) for the QTL located on chromosome arm 2AS and co‐localized with *WheatPME1* gene encoding pectin methylesterase enzyme (Lionetti et al., 2015). This enzyme modulates the degree and patterns of cell wall methyl‐esterification making pectin less susceptible to degradation by pectin degrading enzymes produced by fungal pathogens (Volpi et al., 2013). Pectin content and methyl‐esterification in grasses have largely been associated with plant resistance to pathogens (Volpi et al., 2013; Wiethölter et al., 2003). The co‐localization of *WheatPME1* with the QTL controlling FHB resistance was reinforced by expression analysis in the parental lines of an RIL population segregating for FHB resistance and generated by crossing a resistant hexaploid line deriving from Sumai‐3 with a susceptible durum wheat cultivar (Lionetti et al., 2015). It has been suggested that during infection, resistant plants downregulate *WheatPME1* expression to ensure a higher degree of cell wall methylation, which would protect the cell wall against *Fusarium* pectic enzymes. Similarly, Garvin et al. (2009) mapped a QTL on chromosome 2A of the *T. turgidum* ssp. *dicoccoides* line “Israel A” that increased FHB susceptibility.

On top of already discovered FHB or DON resistance or susceptibility genes, for an even higher number of major or minor QTL involved in FHB or DON resistance, the functions remain unknown so far which does not preclude their deployment in breeding either through phenotypic or genomic selection.

### 4.3 QTL projection on Svevo reference genome

To summarize and compare currently postulated genetic factors contributing to FHB resistance in bread and durum wheat, we projected a total of 642 QTL previously reported in the literature onto the reference genome of durum wheat cv. Svevo at the megabase resolution level. This includes 287 QTL originally mapped in durum wheat and 355 QTL mapped in bread wheat, hence providing valuable insights on the complexity of the wheat QTLome for *Fusarium* resistance and how to best leverage it for breeding purposes.

QTL‐projection on consensus linkage maps and reference genomes enables the comparison and analysis of data from studies that differ in several aspects, such as mapping population, number of lines, and number and type of molecular markers (Salvi & Tuberosa, 2015; Venske et al., 2019). The analysis was conducted following the approach by Veyrieras et al. (2007) and Zheng et al. (2021). Where the confidence interval (CI, 95%) for the QTL was not reported, it was calculated using the Darvasi and Soller (1997) and Liu et al. (2009) formulas. The 642 QTL associated with FHB response, from both linkage analysis (188 QTL) and genome‐wide association studies (GWAS, 454 QTL), were all projected onto the durum wheat genome, using the iSelect Wheat 90K single nucleotide polymorphism (SNP) array from the consensus map of Maccaferri et al. (2015) and the cv. Svevo v.1.0 reference genome (Maccaferri et al., 2019). The tetraploid and hexaploid QTL signals projected onto the Svevo genome are depicted in Figure 1, and their features are listed in Supporting Information 3.

FIGURE 1Quantitative trait loci (QTL) projection on the durum wheat consensus map available in Maccaferri et al. (2015). QTL from durum wheat publications are in yellow, QTL from bread wheat publications are in green. Genes related to Fusarium head blight (FHB) resistance are highlighted in red, and major QTL are highlighted in black. QTL reported in the same publication are aligned vertically.

**Figure:**
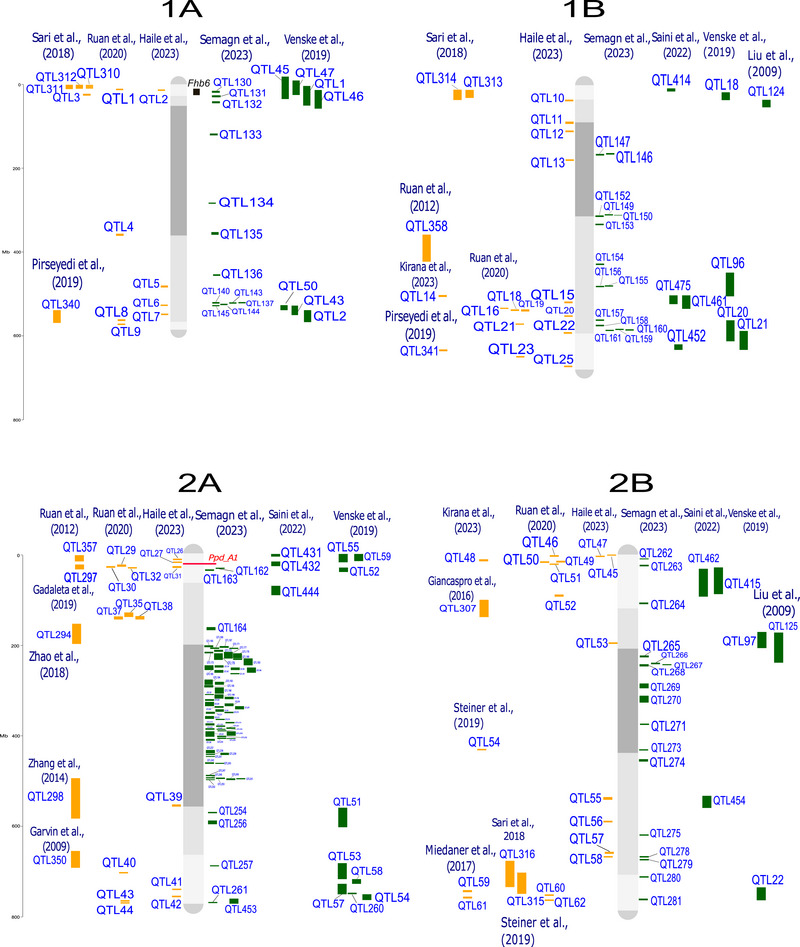

**Figure:**
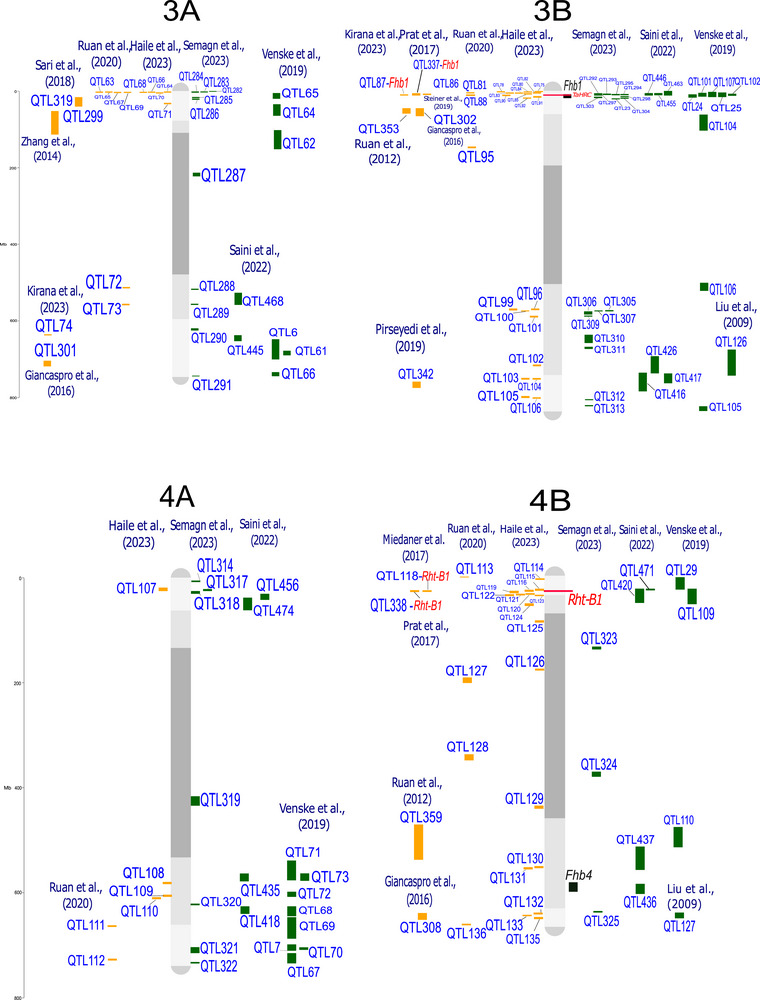

**Figure:**
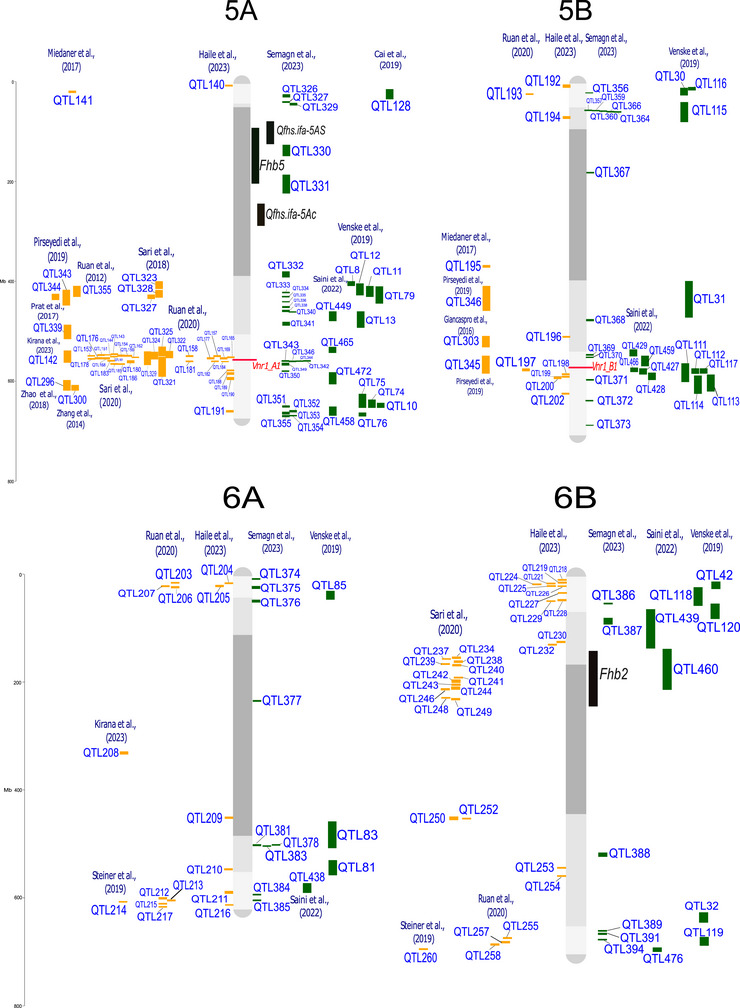

**Figure:**
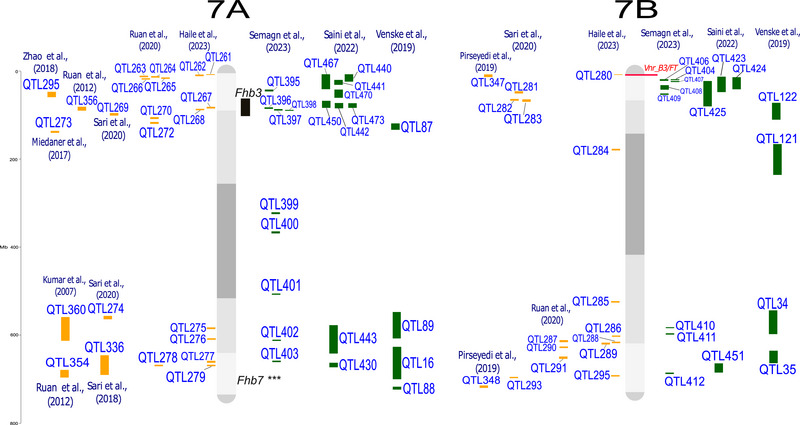

Regarding the different FHB response mechanisms, QTL were categorized as follows: (i) 209 signals for type‐I resistance/FHB incidence; (ii) 228 signals for type‐II resistance/FHB severity; (iii) 126 signals without distinction between type‐I and type‐II (FHB index); (iv) 56 signals for mycotoxin, mainly DON, accumulation; and (v) 20 for FHB‐damaged kernels. These results indicate that loci underlying type‐I and type‐II resistance are present in balanced numbers and equally contribute to the overall resistance. Interestingly, a relatively high number of QTL have been specifically identified for DON content in the kernel, which is of outermost relevance for breeding. Mesterhazy (2020) suggested that low mycotoxin accumulation should be considered the only trait relevant for a more effective selection of FHB resistance.

QTL detected from a range of studies are expected to overlap due to the repeated detection of the same locus across different mapping populations and germplasm. This is particularly true when the parents of mapping populations and genotypes used in GWAS analysis show a certain degree of co‐ancestry, leading to the presence of chromosome segments that are identical‐by‐descent among genotypes. For example, the *Fhb1* locus has been repeatedly identified in different mapping populations and GWAS (Kirana et al., 2023; Prat et al., 2017; Venske et al., 2019). By considering QTL signals from diverse and independent studies in a unique framework, it is possible to identify non‐redundant QTL corresponding to unique loci. This can be achieved by grouping overlapping QTL signals into putative unique QTL clusters according to the region‐specific linkage disequilibrium extent. Out of 642 initial QTL signals, we identified 330 putatively independent QTL signals, including 108 QTL clusters and 222 QTL singletons (detailed in Supporting Information 4), hence highlighting the complexity of the QTLome of FHB resistance in wheat.

A few more than half of QTL clusters (59 out of 108) were composed of both durum wheat and bread wheat colocalized QTL signals, while 28 clusters were bread wheat‐specific and 21 were durum wheat‐specific. QTL clusters with both durum and bread wheat co‐localized QTL signals should be considered loci whose contrasting alleles are present in both species.

## 5 CURRENT STATE OF BREEDING FOR FHB RESISTANCE IN DURUM WHEAT

Over the last two decades, in major durum wheat‐growing regions in North America and Europe, a main target in breeding has been the development of productive and high‐quality durum wheat varieties with improved FHB resistance. However, durum varieties with a high level of FHB resistance are not yet available, and the simple transfer of some highly effective QTL, such as *Fhb1* and *Fhb5* from bread to durum wheat varieties endowed with the necessary adaptability, productivity, and end‐use quality, has been very difficult. As resistance is highly affected by genetic background, the lines with acceptable adaptability and end‐use quality released after backcrossing often lack high and satisfactory FHB resistance due either to linkage drag with undesired traits or the interaction of the recipient genetic background with the expression of loci such as *Fhb1* and *Fhb5* (Hermann Buerstmayr, unpublished results).

In the United States, North Dakota has been a hotspot for FHB outbreaks since 1991 (M. P. McMullen et al., 1997; M. McMullen et al., 2012), with ∼55% of the US durum wheat crop being cultivated in this region (Elias et al., 2021). In response to the threat posed by FHB, the North Dakota durum breeding program has developed nine durum cultivars with increased FHB resistance following a classical pedigree method based on phenotypic selection of elite progenies derived from crosses among existing varieties and breeding lines. The new cultivars outperform their parental varieties, and all lines developed before 2005 in terms of FHB resistance (Beres et al., 2020; Ransom et al., 2019, 2020), a finding that is believed to result from accumulation of minor, mostly unknown QTL from native germplasm. The cv. Divide (Elias & Manthey, 2007) was the first cultivar with a moderate level of FHB resistance in the region and had been the leading cultivar grown in North Dakota from 2009 to 2016. The recently released ND Riveland has even lower FHB disease severity and DON levels when compared with all other cultivars grown in North Dakota (Elias & Manthey, 2019), and it is now the most popular durum wheat cultivar in the region. The Canadian durum line DT764, which is moderately resistant to FHB (Clarke et al., 2010), is in the pedigree of ND Riveland, therefore ND Riveland likely combined the resistance from DT764 and adapted germplasm in North Dakota.

Recently, the *Th. elongatum Fhb7* locus transferred into bread wheat 7BL (W. Zhang, Danilova, et al., 2022) is being incorporated into adapted spring and winter durum genotypes through a marker‐assisted backcross breeding pipeline in USDA‐ARS (F. Wang et al., 2023) providing breeders with a new source of FHB resistance.

In Canada, durum is grown in Saskatchewan (82% of production) and in Alberta (18%) because the increased FHB pressure in other Canadian regions pushed durum production into the southern prairies of western Canada. Durum wheat cultivars such as Brigade (Clarke et al., 2009), Transcend (Singh et al., 2012), CDC Credence (Pozniak, Clarke, Haile, et al., 2020), and CDC Defy (Pozniak, Clarke, & Haile, 2020) show improved FHB resistance relative to other elite durum wheat cultivars, which were rated as moderately susceptible compared to common wheat checks. Cvs. Brigade and Transcend show a similar level of FHB resistance as ND Riveland.

In 2016, western Canada, including Saskatchewan and Alberta, experienced the most severe FHB epidemic ever, which caused millions of dollars loss in revenue to Canadian durum farmers. Since then, durum breeding programs have significantly increased FHB phenotyping capacities by approximately three times with disease pressures ranging from low to high level, which allow to discriminate from subtle to accumulating differences in FHB resistance derived from the recombination of minor native QTL/genes (Ruan et al., 2020; Sari et al., 2020) through the evaluation of the high number of breeding lines. In 2021, AAC Schrader, the first durum cultivar rated as the intermediate FHB resistance relative to common wheat, was released by Agriculture and Agri‐Food Canada, followed by CDC Wiseton released by University of Saskatchewan in 2023, and DT2033 released by AAFC in 2024 all with intermediate FHB resistance level. The North American germplasm was also used in breeding programs in French leading to a series of FHB tolerant cultivars such as Neodur and Joyau (Trottet et al., 2014).

FHB resistance in durum wheat has not been a priority in CIMMYT's durum breeding program, due to the disease's relative absence in CIMMYT‐mandated areas in the Global South. However, climate change, the expansion of maize‐wheat rotations, and the widespread adoption of conservation agricultural practices have made FHB occurrences in durum more common in East Africa, North Africa, Latin America, and South Asia. Consequently, the importance of FHB resistance as a target trait has grown significantly within CIMMYT's durum breeding program. Initially, a large panel of elite durum lines and landraces was evaluated in the field with negligible success (X. He et al., 2013). Then, based on the positive results on utilization of *Fhb1* in durum wheat (Prat et al., 2017), the relevant QTL haplotype has been introduced into CIMMYT durum germplasm via crosses with a Chinese donor bread wheat line Wuhan‐3 using marker‐assisted selection. However, progenies confirmed to carry the *Fhb1* gene using the molecular marker *Xsnp3BS‐8* (Bernardo et al., 2012) did not outperform their CIMMYT durum parents in terms of FHB resistance, both in field and greenhouse experiments.

Recently, two inhibitors of the *Fhb1* gene have been reported in bread wheat (X. Li et al., 2023). These inhibitors act additively, with the presence of one inhibitor significantly reducing the effect of *Fhb1*, and both inhibitors substantially masking the effect of Fhb1. Using linked markers, the Fhb1‐carriers CIMMYT durum lines were tested for the two inhibitors. Results revealed that all of them carried the second inhibitor (*Fhb1‐In2*), while half of them had the first one (*Fhb1‐In1*). This may explain why *Fhb1* did not contribute to FHB resistance when introduced into CIMMYT durum lines. Subsequent testing of a panel of 175 durum lines from India showed that 63% had *Fhb1‐In1* and 89% had *Fhb1‐In2*, with only 12 lines lacking both the inhibitors, indicating a widespread presence of *Fhb1* inhibitors. With this new information, speed breeding facilities at CIMMYT will be utilized to eliminate the two inhibitors in *Fhb1*‐carrying breeding lines and other elite backgrounds. Progenies will be compared to their CIMMYT parents to assess if the elimination of inhibitors improves FHB resistance, and breeding lines exhibiting good FHB resistance will be identified for future breeding activities.

In Europe, the durum production zone is expanding beyond the traditional summer‐dry Mediterranean region into more humid central and eastern Europe, which is more prone to FHB epidemics. While most durum wheat is grown as spring types, often planted in autumn, the more continental part of Europe has traditionally grown spring types sown in early spring. FHB resistance is needed for all durum wheat‐growing regions and cultivar types, especially in regions with higher humidity and precipitation during flowering and grain filling; nevertheless, even the most modern cultivar releases remain susceptible to FHB.

Research efforts during the recent 20 years have led to stepwise increase of FHB resistance in pre‐breeding material adapted to European conditions. Successful deployment of *Fhb1* (Prat et al., 2017), a few accessions of wild (Buerstmayr et al., 2013; Gladysz et al., 2007) and domesticated emmer wheat (Huber et al., 2008), and quantitative variation in the adapted durum wheat pool (Miedaner & Longin, 2014; Moreno‐Amores, Michel, Miedaner, et al., 2020; Steiner, Michel, et al., 2019; Talas et al., 2011) led to improved experimental lines, which are available for the next breeding cycle in cultivar development programs (Kirana et al., 2023). Recent data show that an increased anther extrusion in durum wheat is highly correlated with increased FHB resistance, and therefore this trait can be considered for indirect selection (Barbara Steiner, unpublished). Different from bread wheat, a potential issue associated with the deployment of the anther extrusion escape trait in durum could be related to a potential increase in drought susceptibility, with negative consequences for the overall spike fertility. Combining improved germplasm with alien introgressions, such as *Fhb7E* or *Fhb7el_2_*, improved lines from other breeding programs, and selecting for increased anther extrusion is expected to further enhance FHB resistance in the regionally adapted durum wheat gene pools, although breeders are often reluctant to deploy non‐adapted exotic alleles for cultivar development. A possible solution relies on quantitative variation for FHB resistance in the regionally adapted gene pool, a strategy that requires several breeding cycles as QTL in native material typically have rather small effects to be adequately detected.

In Italy, both native North American and French germplasm carrying multiple QTL for quantitative resistance as well as *Fhb1*‐ and *Fhb5*‐introgression lines have been repeatedly used in breeding. This process has been followed by effective selection under high disease pressure, including mist‐irrigation and artificial inoculation. These efforts led to the release of some durum wheat cultivars with improved FHB tolerance as compared to the core set of native, highly susceptible germplasm. Furthermore, from crosses and top‐crosses between durum wheat segmental introgression lines carrying either *Fhb7el_2_* or *Fhb7EL* on durum wheat 7AL (Kuzmanović et al., 2021) and several durum varieties adapted to Mediterranean environments but lacking any defense against *Fusarium* diseases, advanced breeding lines are being produced using *Thinopyrum*‐specific, user‐friendly markers (Carla Ceoloni and Ljiljana Kuzmanović, unpublished).

## 6 IMPACT OF CROP MANAGEMENT ON SPREAD OF FHB IN DURUM WHEAT

Whenever genetic resistance is insufficient to control FHB, crop management strategies can be adopted to minimize the impact of the disease. The most popular approaches involve agronomic practices that limit/reduce the amount of inoculum in the field as well as protocols for chemical or biological control (Shah et al., 2018). The efficient early detection and identification of causal pathogens are also essential to adopt effective management practices that reduce or prevent their spread in order to mitigate the disease.

The adoption of proper agronomic practices may help to reduce FHB outbreaks. The rotation of wheat with a non‐host crop species such as soybean is highly recommended to avoid the risk of disease development and DON accumulation even though some studies have demonstrated that *F. graminearum* can also colonize soybean and become a source of inoculum load in the field (Chiotta et al., 2020; Kang et al., 2019). Besides a proper tillage technique to reduce crop residues, the removal of non‐cultivated plants such as weeds, including other grasses, is advised as they may represent reservoir hosts for FHB pathogens (Fulcher et al., 2019; Suproniene et al., 2019). Moreover, it has been reported that advancing sowing date and proper irrigation system to manage the moisture in the fields are important for reducing the development of the disease and the DON level in wheat (Cowger et al., 2009). As it is difficult to consider planting as a management date because of the unpredictable weather patterns from year to year (Wiersma et al., 1996), it is currently recommended to plant across a planting window up to 2 weeks after the optimal date to avoid disease risk (Friskop et al., 2018). Jbir et al. (2022) found that late planting was able to reduce FHB symptoms without any impact on DON levels. The type and amount of fertilizers are also correlated with the concentration of mycotoxins (Yi et al., 2001; Podolska et al., 2017). Managing FHB outbreaks through agricultural practices is a challenge for growers especially when the climatic conditions are favorable for infection, so more effective option tools are needed to incorporate into the integrated FHB management strategy. Recently, it has been reported that the amendment of silica to the soil as fertilizer, especially when combined with fungicide treatments, is effective in reducing disease in wheat (Pazdiora et al., 2022, 2021; Sakr, 2021). However, there is a debate that need to be clarified as two recent reports showed that applications of silica did not reduce the fungal growth and that mycotoxin contamination in kernels was even more severe in susceptible cultivars (Pazdiora et al., 2022; Sakr, 2021). As much as some management approaches ensure certain levels of reduction in FHB severity and mycotoxin contamination, no single control strategy will provide significant control of FHB. Therefore, there is a pressing need to incorporate more effective options into the integrated FHB management strategy.

The use of fungicides remains the major tactic for a better FHB control. The most used fungicides are demethylation inhibitors (DMI) and quinone outside inhibitors (QoI). The optimum timing of fungicide application for successful control of FHB is during the 6‐day window starting at early anthesis (Bolanos‐Carriel et al., 2020; Freije & Wise, 2015). Applications made too early or too long after anthesis are not recommended as their efficacy in controlling FHB and DON contamination during disease favorable growing seasons is too low (Paul et al., 2018). Technologies such as remote sensing can detect first signs of FHB, allowing targeted and timely application of fungicides, thereby minimizing prolonged and intense fungicide exposure, which may help to avoid the emergence of fungicide‐resistant populations (Xiao et al., 2022; H. Zhang, Huang, et al., 2022).

Since chemical fungicides can potentially pose adverse effects on human and/or the environment (Maltby et al., 2009; Zubrod et al., 2019), the integration of biocontrol agents may represent an effective support. Numerous fungi and bacteria acting against *Fusarium* spp. were identified. Inhibitory interactions between host and pathogen can be direct, most commonly, by hindering the fungal growth and suppressing disease progress (Matarese et al., 2012; Wachowska & Glowacka, 2014; A. G. Xue, Chen, Sant'anna, et al., 2014; A. G. Xue, Chen, Voldeng, et al., 2014) or through antagonistic compounds (Oufensou et al., 2020; Malbrán et al., 2020) such as antifungal metabolites (Hao et al., 2021). An indirect inhibition can be manifested through the stimulation of plant defense responses by microbes colonizing the host or rhizosphere. This can occur via the release of volatiles and phytohormones or by improving the host nutrient acquisition capacity (Adnan et al., 2022; Ilyas & Bano, 2012; Jha, 2020; Qu et al., 2020; Vandana et al., 2021).

Many studies have documented the direct effect of several fungi on FHB pathogens including *Trichoderma* spp., *Sphaerodes mycoparasitica*, *Clonostachys rosea*, *Aureobasidium pullulans*, and *Cryptococcus* spp. (A. He et al., 2019; Vujanovic & Goh, 2009; Z. Zhao et al., 2014). In addition to fungi, several bacterial agents including *Pseudomonas* spp., *Lysobacter enzymogenes*, *Bacillus* spp., and *Streptomyces* spp. have also been described to exhibit an antagonistic activity against FHB pathogens (Jochum et al., 2006; Palazzini et al., 2007; Schisler et al., 2006, 2002; Zanon et al., 2024; Z. Zhao et al., 2014). Mycoviruses in *F. graminearum* have also been reported to influence the fungal metabolism and reduce the disease severity (Bormann et al., 2018; Darissa et al., 2012).

Besides antagonistic effects, several surveys have characterized microbial candidates able to degrade, adsorb, or convert fungal mycotoxins into less toxic metabolites. Examples include the bacterial genera *Nocardioides* and *Devosia*, which are present in the wheat phyllosphere and rhizosphere (Wachowska et al., 2017; H. Zhang, Zhang, et al., 2021). The application of microbial candidates via seed coating (Mattei et al., 2022), spraying on wheat heads (Baffoni et al., 2015), or soil drenching (Elnahal et al., 2022) proved to be an effective management strategy against FHB; however, the main challenge is to design and develop formulations that are highly effective and easily used with a long shelf‐life.

## 7 HOW TO ACHIEVE A HIGH‐LEVEL FHB RESISTANCE IN DURUM WHEAT?

Although the recently released durum varieties show some FHB resistance, their resistance levels are still not comparable to resistant common wheat varieties either carrying major QTL such as *Fhb1* or based on quantitative FHB resistance. In general, durum wheat varieties with the highest level of FHB resistance are only comparable to common wheat varieties rated as moderately susceptible and can perform well under low and moderate disease pressures but are still vulnerable to severe FHB epidemics. To further improve FHB resistance in durum wheat, a combination of complementary approaches should be considered. Here, we highlight the priority actions that should be implemented to achieve satisfactory resistance to FHB in durum wheat.

### 7.1 Providing accurate diagnosis and identification of involved pathogen

Supporting additional pathogen surveillance is critical for the detection, monitoring, and managing diverse *Fusarium* populations that may cause FHB in durum wheat. In recent years, new fungal chemotypes, more aggressive pathogen species and genotypes, and emergence of resistance to fungicide have been observed (Bamforth et al., 2022; de Chaves et al., 2022; A. C. Kelly et al., 2015). While significant pathogen and toxin surveillance exists for bread wheat, less is available for durum wheat, necessitating additional monitoring to understand the scope, scale, and risks of FHB in this crop. Increasing the amount of surveillance is important, using common sample and data collection methods can also enhance the impact.

One recommendation would be the transition toward more standardized culture and data collection and depositing. Historically, pathogen identification was performed using microscopy analysis of colony and spore morphology; however, low‐cost whole genome sequencing is now widely accessible and valuable, particularly when sequences are deposited in international repositories such as NCBI/GenBank (https://www.ncbi.nlm.nih.gov/genbank/) and ENA (https://www.ebi.ac.uk/ena/browser/home). Additionally, standardization testing for toxins, particularly the trichothecene chemotypes (i.e., 15‐ADON, 3‐ADON, NIV, T‐2, HT‐2, and NX), and toxin variants that may occur during host toxin detoxification or food processing (i.e., DON‐3‐glucoside) could also provide significant benefit. This is increasingly important, based on recent fungal community screening of durum wheat fields worldwide, which reports increased incidence of less aggressive species, such as *Fusarium poae* (*Sambucinum* clade of FSAMC), members of the *Fusarium tricinctum* species complex (FTSC), and *Fusarium proliferatum* (a member of the *Fusarium fujikuroi* species complex, FFSC). Additionally, within FSAMC, other species such as *F. sporotrichioides* and *F. langsethiae* (*Sporotrichioides* clade) are considered important members of the *Fusarium* community (Senatore et al., 2023, 2021).

When possible, isolates should be deposited in a recognized national or international repository, such as CBS in Europe (https://wi.knaw.nl/Collection), NRRL collection in the United States (https://nrrl.ncaur.usda.gov/), and DAOMC collection in Canada (https://agriculture.canada.ca/en/science/collections/canadian‐collection‐fungal‐cultures‐daomc). This will ensure that physical specimens linked to other metadata, such as genomics information, are available to researchers and breeders for validating results and trends across fungal species and populations. However, regional differences in plant pathogen regulations may limit or restrict the transport and use of isolates, particularly when imported/exported. Scientists must be aware and comply with their regional regulations to limit the spread of new or emerging isolates that may not be present in some regions.

Wild grasses serve as reservoir for *Fusarium* infections and mycotoxin contamination of wheat, which pinpoints the importance of understanding the native biology of the *Fusarium* species complex (Gerling et al., 2023). We therefore argue that there are still large gaps in our basic understanding of FHB species complex biology, ecology, genetics, and epidemiology hindering our capacity to develop novel management strategies. Therefore, investigating the microbiome drivers of FHB complex assembly, the ecological roles of *Fusarium* mycotoxins, and the contribution of wild hosts to *Fusarium* population fitness and epidemiology is essential.

With specimens available, breeders and pathologists can carefully select isolates for inoculation in their disease studies and screening. Careful consideration should be made when selecting isolates as part of disease nursery design since it is known that the pathogen will interact differently across host species and genotypes (Ruan et al., 2021). Likewise, isolate–isolate interactions may occur and cause interference if multiple isolates are used and combined, which may or may not be desired as part of the disease nursery design (Walkowiak et al., 2015). While interactions between isolates, hosts, and environment are crucial for determining disease outcomes and toxin accumulation, this area requires more systematic study as these factors likely contribute to the high variability often observed in *Fusarium* testing data between test sites and years.

### 7.2 Marker‐assisted selection for pyramiding FHB resistance loci

Marker‐assisted selection is a useful breeding strategy to trace and select for a limited number of large effect QTLs. As the most effective loci have been identified in bread wheat or related species, intense pre‐breeding activity is needed to transfer these loci into durum lines through marker‐assisted selection. Some lines with single or multiple loci are already available, and these can be used to pyramid different resistance genes into the same genotype.

Although the extent of resistance due to *Fhb1* in durum wheat was variable depending on the recipient backgrounds (Giancaspro et al., 2016; Kirana et al., 2023; Prat et al., 2017), the locus has shown overall efficacy, even in the absence of the D genome, in conferring a largely prevailing type‐II resistance to durum wheat. Combining *Fhb1* and *Qfhb.rwg‐5A.2* (Chu et al., 2011; M. X. Zhao et al., 2018) with other major loci such as the alien *Fhb7* appears an attractive possibility for creating a more robust resistance in different varietal backgrounds. The defense mechanisms underlying the *Fhb1*‐*Fhb7* combination are particularly effective in terms of resistance for spreading (type‐II resistance) and reduction of DON production (e.g., Buerstmayr et al., 2021; Fanelli et al., 2023). However, while the degree of expression of these mechanisms is higher in the presence of *Fhb7* versus *Fhb1*, with no significant dependency on the recipient genotype and yield penalty for the former as observed for *Fhb1* alone (e.g., Kuzmanović et al., 2021, 2019; Prat et al., 2017), no major increment in the resistance response seemed to be conferred by *Fhb1+Fhb7* in the genotypes where the assembly was so far developed (Gyawali et al., 2023; Shen & Ohm, 2006).

The combination of *Fhb4*, *Fhb5*, and *Fhb1* in Chinese bread wheat germplasm led to a strong type‐I resistance, along with significant improvement in type‐II resistance (Y. Zhang, Yang, et al., 2021). Nevertheless, although *Fhb5* has been shown to enhance type‐I resistance in bread wheat, the development of stable durum wheat lines has not been successful. Durum lines homozygous for *Fhb5* showed a strong tendency toward sterility (Hermann Buerstmayr, unpublished results).

The *Fhb7*‐based resistance remains unique, not only for its efficacy against the FHB disease, but also for its ability to protect against Fusarium crown rot. Effectiveness toward both diseases, assayed in bread and durum wheat through infection with different *Fusarium* species (*F. graminearum*, *F. culmorum*, and *F. pseudograminearum*; Ceoloni et al., 2017; Kuzmanović et al., 2019), is an exceptional attribute of *Fhb7*. This coincidence is particularly profitable for durum wheat, the most susceptible among small‐grain temperate cereals and largely cultivated in semi‐arid regions (e.g., in northern Africa, southern Europe and Australia) where environmental and climatic conditions greatly favor FCR attacks (Alahmad et al., 2020; Chekali et al., 2016).

Several QTL controlling resistance to FHB are available in the durum wheat genetic background. For instance, resistance QTL carried by durum cultivars and breeding lines from Canada and Austria introgressed from Sumai‐3, wild emmer, or domesticated emmer have been described in recent studies by Ruan et al. (2020) and Haile et al. (2023). A meta‐analysis grouping all QTL for reaction to FHB identified in bread and durum wheat is presented in Figure 1. This inventory is particularly helpful in selecting the hotspot regions that do not co‐locate with the *Rht* alleles known to be negatively correlated with resistance to FHB (Supporting Information 2 and 3). In addition, a detailed list of QTL related to FHB/DON resistance, along with potentially resistant lines aimed at improving FHB resistance specifically in durum wheat, is reported in Table 3.

**TABLE 3:** List of reported quantitative trait loci (QTL) for Fusarium head blight (FHB)/deoxynivalenol (DON) resistance and potential resistant lines used in durum wheat breeding program (updated from Haile et al., 2019).

No.	FHB resistance source	Remark	DNA marker	QTL reported Chr.	Reference	Affiliated/source institute
1	FA‐15‐3 (syn. “Israel A”)	Chr 3A of *T. turgidum* ssp. *dicoccoides* carries resistance gene(s) to head bleaching due to FHB	*Xgwm2*	3A	Ban and Watanabe (2001)	Japan Int. Res. Center for Agri. Sciences (JIRCAS), Tsukuba, Japan // Ban T: tomohiro@affrc.go.jp
2	83 RICL individuals	In a Langdon‐16 durum background, the resistant recombinant inbred chromosome lines that harboring *Qfhs.ndsu‐3AS* will be useful for the introgression of this QTL to adapted wheat backgrounds	*Xgwm2, Xfcp397.2*	3A	Chen et al. (2007); Otto et al. (2002)	University of Minnesota, Dept. of Agronomy and Plant Genetics, St. Paul, USA Dept. of Plant Sciences, North Dakota State University, Fargo, USA // Xiwen Cai: xiwen.cai@ndsu.edu
3	DT696 Blackbird	DT707 × DT696 Strongfield × Blackbird	*Xwmc474* *Xgwm55* *Xgwm518*	5A 2BL (from Strongfield) 6BS (from Blackbird)	Sari et al. (2018) Somers et al. (2006)	Agriculture and Agri‐Food Canada, Swift Current Research and Development Centre, Swift Current, Canada
4	Mt.Hermon#22	*T. turgidum* ssp. *dicoccoides* line from the collection of the Institute of Evolution, University of Haifa, Israel	*Xgwm2* *Xgwm610* *Xbarc167* *Xgwm375*	3A 4A 2B 4B	Gladysz et al. (2007)	Institute of Biotechnology in Plant Production, BOKU‐ University of Natural Resources and Life Sciences Vienna, Tulln, Austria // Hermann Buerstmayr: hermann.Buerstmayr @boku.ac.at
5	123 RILs Euploid (LDN × PI478742 7A)	Langdon (LDN) has a pair of *T. turgidum* sp. *dicoccoides* PI478742 7A chromosomes substituted for the native 7A chromosomes (LDN‐DIC 7A)	*Xbarc121* (detected a 210‐bp LDN, may be of use for marker‐assisted selection)	7A	Kumar et al. (2007)	North Dakota State University, Department of Plant Sciences, Fargo, USA // Justin D Faris: farisj@fargo.ars.usda.gov
6	ND2710 Tun18 Tun36 Tun108 Tun134	Tetraploid wheat sources of resistance from Tunisia selected among many lines evaluated over five repeated FHB trials	*wPt‐0054* *wPt‐7279* *wPt‐2885* *wPt‐6910*	5BL	Ghavami et al. (2011); Huhn et al. (2012)	North Dakota State University, Department of Plant Sciences, Fargo, USA // Shahryar Kianian: s.kianian@ndsu.edu; Elias: elias.elias@ndsu.edu
7	Divide Tun7	North Dakota State University cultivar and partially resistant. Selected from the cross “Ben” (PI596557)/D901282//“Belzer” (PI603286) Tunisian landrace (tall, late maturing, and adapted to drought‐prone growing conditions)			Fakhfakh et al. (2011b)	Laboratory of Plant Breeding, National Agronomy Institute of Tunis (INAT), Tunis‐ Mahrajene, Tunisia // Fakhfakh M: fmedmoez@yahoo.fr North Dakota State University, Dept. of Plant Sciences, Fargo, USA // Shahryar Kianian: s.kianian@ndsu.edu
8	DS × Td161 Floradur × Td161 Helidur × Td161	Fusarium‐resistant homozygous *T. turgidum* spp. *dicoccum* line by crossing with three susceptible durum wheat varieties, three populations segregating for FHB resistance were generated	*Xwmc398* *Xgwm132* *Xgwm400* *Xwmc617* *Xbarc133*	6B 6A 7B 4B 3B	Buerstmayr et al. (2012)	Institute of Biotechnology in Plant Production, BOKU‐ University of Natural Resources and Life Sciences Vienna, Tulln, Austria // Hermann Buerstmayr: hermann.Buerstmayr @boku.ac.at
9	BGRC3487 × 2*DT735	RILs	*wPt‐6239* *wPt‐7076*	3B 7A	Ruan et al. (2012)	Department of Plant Sciences, Crop Development Centre, University of Saskatchewan, Saskatoon
10	Mt. Gerizim #36	*T. turgidum* spp. *dicoccoides* line with moderate FHB resistant. It is a hulled wheat with brittle rachis, has a short and awned spike phenotype, tough glumes, and is tall and sensitive to lodging	*Xgwm626*	6B	Buerstmayr et al. (2013)	Institute of Biotechnology in Plant Production, BOKU‐ University of Natural Resources and Life Sciences Vienna, Tulln, Austria // Hermann Buerstmayr: hermann.Buerstmayr @boku.ac.at
11	Logidur and Wintergold DGE‐1	Commercial winter durum wheat varieties (mean FHB < 5) Disomic addition line (mean FHB < 3)			Miedaner and Longin (2014)	State Plant Breeding Institute, University of Hohenheim, 70593 Stuttgart, Germany // Carl Friedrich Horst Longin: Friedrich.Longin@uni‐hohenheim.de
12	BP025 population (Ben and PI 41025)	PI 41025 is a cultivated emmer wheat (*T. turgidum* spp. *dicoccum*) accession and moderately resistance to FHB	*IWA1103, IWA111* *Xwmc110, IWA7009* *IWA7649, IWA5039*	2A 5AL 3A	Q. Zhang et al. (2014)	Crop Improvement and Genetics Research Unit Western Regional Research Center, USDA ‐ Agricultural Research Service, Albany, CA, USA // Steven Xu: steven.xu@usda.gov
13	02‐5B‐318	02‐5B‐318 is an FHB‐resistant accession derived from Sumai‐3 crossed with the durum wheat cv. Saragolla (FHB‐susceptible). Each progeny was evaluated for the presence or the lack of D genome chromosomes by using a set of 14 single bands, D genome‐specific gSSR markers, and one mapping on the short and one on the long arm of each D genome chromosome	*IWB63138* *IWA1721* *IWB43304* *IWB37509* *IWB64332* *IWB72334* *IWB65943* *IWB55365* *IWB48353* *IWB816*	2AS 6B 7AL 3AL 3BS 5BL 1BL 2BS 4BS 5BS	Giancaspro et al. (2016)	Department of Soil, Plant and Food Sciences, University of Bari Aldo Moro, Bari, Italy // Agata Gadaleta: agata.gadaleta@uniba.it
14	Karur × DBC‐480	Carries an Fhb1 introgression from Sumai‐3. Line developed by four generations of marker‐assisted selection‐backcrossing of Sumai‐3 into the background of the Austrian durum variety Semperdur and subjected to rigorous phenotypic selection for FHB resistance	*Xbarc147* *Xumn10*	3B 3B	Prat et al. (2017)	Institute of Biotechnology in Plant Production, BOKU‐ University of Natural Resources and Life Sciences Vienna, Tulln, Austria // Hermann Buerstmayr: hermann.Buerstmayr @boku.ac.at
15	10Ae564	An introgression durum line with PI 277012 resistance (PI 277012 is a bread wheat line with a high level of FHB resistance across different environments)	*IWB26525* *IWB74024*	5A 7A	M. X. Zhao et al. (2018)	Crop Improvement and Genetics Research Unit Western Regional Research Center, USDA ‐ Agricultural Research Service, Albany, CA, USA // Steven Xu: steven.xu@usda.gov
16	Ben × Tunisian 108	Backcross inbed lines	*wpt‐1818* *wpt‐7975*	1B 7B	Pirseyedi et al. (2019)	Department of Plant Sciences, North Dakota State University, Fargo, ND, 58108, USA // Elias Elias: Elias.Elias@ndsu.edu
17	International collection of 228 genotyped durum wheat cultivars	Diverse panel	*IWB72690* *IWB46663* *IWB5439* *IWB64968* *IWB70133* *IWB66697*	1A 2A 2B 3B 6A 6B	Steiner, Michel, et al. (2019)	Institute of Biotechnology in Plant Production, BOKU‐ University of Natural Resources and Life Sciences Vienna, Tulln, Austria // Sebastian Michel: sebastian.michel@boku.ac.at
18	Diverse durum wheat (*Triticum turgidum* L.)	Elite Canadian cultivars, advanced breeding lines, recently developed germplasm from Canadian breeding programs and experimental durum lines representing exotic FHB resistance and germplasm from global collections	*BS00083459_51* *CAP11_c6014_160* *RFL_Contig399_1148* *BS00021990_51* *wsnp_JD_c6331_7499499* *CAP12_c1085_283* *wsnp_CAP8_c2110_1147974* *Excalibur_c6027_1035* *wsnp_Ra_c10658_17500498* *Tdurum_contig45787_512* *Tdurum_contig15440_616* *JD_c11869_1300* *Excalibur_c20417_743* *BobWhite_c2453_460*	1A 1B 2A 2B 3A 3B 4A 4B 5A 5B 6A 6B 7A	Ruan et al. (2020)	Swift Current Research and Development Centre, Agriculture and Agri‐Food Canada, Swift Current, SK, Canada // Wentao Zhang, Wentao.Zhang@nrc‐cnrc.gc.ca
			*IAAV2383* *Tdurum_contig84762_189* *BS00079522_51* *Kukri_c2074_739* *Tdurum_contig42638_383* *tplb0025f09_1052* *Kukri_c66171_54* *RFL_Contig3368_209* *BS00041063_51* *BS00067701_51* *BS00033182_51* *Jagger_c3477_441* *Kukri_rep_c103067_248* *RAC875_rep_c113337_153* *RAC875_c11969_384* *BS00040600_51* *Kukri_c64387_191*			
19	Canadian and European durum wheat cultivars and breeding lines	Elite Canadian and US cultivars advanced breeding lines and recently developed germplasm from Canadian breeding programs (Crop Development Centre, University of Saskatchewan and Swift Current Research and Development Centre, Agriculture and Agri‐food Canada) and research projects. European *Triticum durum* cultivars and experimental lines developed by single‐seed descent by crossing a resistant tetraploid experimental line DBC‐480 to Karur and Durobonus (susceptible European *T. durum* cultivars) and the advanced breeding line SZD1029K	*Ra_c4159_2716* *BS00000209_51* *Excalibur_c39451_68* *Kukri_c12804_620* *RAC875_c4954_943 wsnp_Ex_c23633_32868822* *TA004185‐0427 RAC875_c5966_1854* *RAC875_rep_c109105_57 Excalibur_c62826_254* *BobWhite_c6462_373* *wsnp_BF482960B_Ta_1_4 RAC875_c27536_611 BS00021984_51 Ex_c101685_711* *Tdurum_contig14562_607* *Ra_c41921_951*	1A 2A (KASP marker) 2B 2B (KASP marker) 3A 3B 3B (KASP marker) 3B 4B 5A (KASP marker) 5B 6A 6B (KASP marker) 7A 7B	Haile et al. (2023)	Department of Plant Sciences, Crop Development Centre, University of Saskatchewan, Saskatoon // jemanesh.haile@usask.ca; curtis.pozniak@usask.ca
			*IAAV3365, BS00075959_51 wsnp_AJ612027A_Ta_2_5 BobWhite_c21949_150 wsnp_BF293620A_Ta_2_1 Kukri_c33022_198* *wsnp_Ra_c24619_34168104* *Ra_c2216_1442* *Ra_c29107_289, Excalibur_c25211_828* *Excalibur_c30648_924 Kukri_c3009_267* *Tdurum_contig45714_427 RAC875_c34994_183* *Tdurum_contig69067_405* *Kukri_c51101_351* *Excalibur_c49736_1197 IAAV3713*			

Abbreviation: MAS, monoacetoxyscirpenol.

This represents a strategic source of information to select QTL, closely linked markers and durum lines carrying useful alleles to design a properly cost‐ and time‐effective breeding program leveraging the most updated approaches such as KASP markers. Accordingly, some markers, consistent across environments and testing models, have been converted into KASP markers and validated using the Global Durum Diversity Panel (Mazzucotelli et al., 2020; Table 4). These include *QFhb‐2A.3*, *QFhb‐2B.4*, *QFhb‐3B.2*, *QFhb‐5A*, *QFhb‐6B.1*, and *QFhb‐6B.3* (Haile et al., 2023). However, many identified QTL are linked with plant height and/or flowering time loci, suggesting that phenology, flowering, and height genes form a complex network that influences FHB resistance in durum wheat (X. He et al., 2016). Therefore, it is recommended to couple testing for FHB resistance with the evaluation of pheno‐morphological traits. Notably, the typical association of short stem with high FHB susceptibility makes the selection of lodging resistant semi‐dwarf cultivars combined with high FHB resistance a challenging task (Haile et al., 2023). As for many durum wheat growing‐regions, semi‐dwarf cultivars are compulsory to ensure lodging resistance and stable yields, the pleiotropic effects on increased FHB susceptibility must be compensated through the selection of short stem and FHB resistant lines. This is feasible as recent results have shown (Kirana et al., 2023).

**TABLE 4:** List of polymerase chain reaction (PCR)‐based markers associated to Fusarium head blight (FHB)‐resistant loci available for durum wheat breeding

No.	QTL (gene)	Marker name	Marker type	Primer name	Primer sequence	Validation population	Reference
1	*Fhb1‐TaHRC*	Fhb1‐TaHRC	KASP	Fhb1‐TaHRC‐S‐FAM‐INS Fhb1‐TaHRC‐R‐HEX‐DEL Fhb1‐TaHRC‐Rev	GAAGGTGACCAAGTTCATGCTTTGTCTGTTTCGCTGGGATG GAAGGTCGGAGTCAACGGATTGCTCACGTCGTGCAAATGGT CTTCCAGTTTCTGCTGCCAT	A diversity panel of 143 common wheat accessions and various durum and common wheat mapping populations	Bonman et al. (2015); Su et al. (2019, 2018)
2	*QFhb‐2A.3*	BS00000209_51	KASP	BS00000209_51_HF BS00000209_51_FF BS00000209_51_KR	GAAGGTCGGAGTCAACGGATTCTGATCCTTGTACAGGGCATTATT GAAGGTGACCAAGTTCATGCTCTGATCCTTGTACAGGGCATTATC CCCATCTTGAAGTCTGGCATG	Global Diversity Panel of tetraploid wheat (Mazzucotelli et al., 2020)	Haile et al. (2023)
3	*QFhb‐2B.4*	2B_Kukri_c12804_620	KASP	2B_Kukri_c12804_620_HF 2B_Kukri_c12804_620_FF 2B_Kukri_c12804_620_KR	GAAGGTCGGAGTCAACGGATTTATAATTGATGACTTATGGGC GAAGGTGACCAAGTTCATGCTTATAATTGATGACTTATGGGA AGAGCAACATCCTCAATTTCC	Global Diversity Panel of tetraploid wheat (Mazzucotelli et al., 2020)	Haile et al. (2023)
4	*QFhb‐3B.2*	RAC875_rep_c109105_57	KASP	RAC875_rep_c109105_57_HF RAC875_rep_c109105_57_FF RAC875_rep_c109105_57_KR	GAAGGTCGGAGTCAACGGATTTGCCGAAGCTGTAAACATCT GAAGGTGACCAAGTTCATGCTTGCCGAAGCTGTAAACATCC GCGGGCTTTTAACGATGCA	Global Diversity Panel of tetraploid wheat (Mazzucotelli et al., 2020)	Haile et al. (2023)
5	*QFhb‐5A*	IAAV3365	KASP	IAAV3365_HF IAAV3365_FF IAAV3365_KR	GAAGGTCGGAGTCAACGGATTCGTGTGCCATTCTCTGAATCATAA GAAGGTGACCAAGTTCATGCTCGTGTGCCATTCTCTGAATCATAG TGCTGGATTTGTTGTGAATTGA	Global Diversity Panel of tetraploid wheat (Mazzucotelli et al., 2020)	Haile et al. (2023)
6	*QFhb‐6B.1*	Kukri_c3009_267	KASP	Kukri_c3009_267_HF Kukri_c3009_267_FF Kukri_c3009_267_KR	GAAGGTCGGAGTCAACGGATTAGGAATGTTGGCCCTCAGCTA GAAGGTGACCAAGTTCATGCTAGGAATGTTGGCCCTCAGCTG ATATACATTGCATTTGGTGGCTCGA	Global Diversity Panel of tetraploid wheat (Mazzucotelli et al., 2020)	Haile et al. (2023)
7	*QFhb‐6B.3*	RAC875_c34994_183	KASP	RAC875_c34994_183_HF RAC875_c34994_183_FF RAC875_c34994_183_KR	GAAGGTCGGAGTCAACGGATTTAAACATGAAAGTCATGGCCT GAAGGTGACCAAGTTCATGCTTAAACATGAAAGTCATGGCCC CTTCTGCTCAAGTGCCTACTT	Global Diversity Panel of tetraploid wheat (Mazzucotelli et al., 2020)	Haile et al. (2023)
8	*Fhb7 (GST)*	KASP‐GST1 (SNP located at 828 bp upstream of the GST start codon)	KASP	KASP‐GST1FAM KASP‐GST1HEX KASP‐GST1R	CTTATAAGGTGGTGCACATCT CTTATAAGGTGGTGCACATCA TTAGTCCCACATGGCTAGTT	RIL population of K114663 × K2620 and RWG34‐NILs	L. Zhao et al. (2022)
9	*Fhb7 (GST)*	KASP‐GST2 (SNP located at 52 bp upstream of the stop codon in the GST coding region)	KASP	KASP‐GST2FAM KASP‐GST2HEX KASP‐GST2R	AGCGCATCATGCAGCTGG AGCGCATCATGCAGCTGC GTGAGTGAGTGGCAGGAG	RIL population of K114663 × K2620 and RWG34‐NILs	L. Zhao et al. (2022)
10	*Fhb7^The2^ (GST)*	Xwgc2318	PACE	Forward 7E‐66‐ FAM Forward 7B‐66‐ HEX Reverse‐66	CCTATGCCGATGTTGTCCTAAAGC ATGCCGATGTTGTCCTAAAGTGCC ATCTGCCGAATGAAAAGAACATGC	Common wheat lines/varieties CS, DS7E(7B), WGC002, PI 277012, Millennium, Wesley, Anton,Mace, Sumai 3, Wangshuibai, and Frontana; Durum line 2021MD1026	Cai et al. (2024)
11	*Fhb7^The2^ (GST)*	Xwgc2319	PACE	Forward *Fhb7^The2^*‐128‐ FAM Reverse *Fhb7^The2^*‐128 Forward 7B‐66‐ HEX Reverse‐66	GGCCACGTATGCGGACAT CCTCCTGCCACTCGCTCAC ATGCCGATGTTGTCCTAAAGTGCC ATCTGCCGAATGAAAAGAACATGC	Common wheat lines/varieties CS, DS7E(7B), WGC002, PI 277012, Millennium, Wesley, Anton,Mace, Sumai 3, Wangshuibai, and Frontana; Durum line 2021MD1026	Cai et al. (2024)
12	*Fhb7^The2^ (GST)*	Xwgc2320	STS	Forward *Fhb7^The2^*‐122 Reverse *Fhb7^The2^*‐122 Forward 7B‐123 Reverse 7B‐123	CTGCTCTTCCCGCTGTCCGAGATT GAACGGCCCGCTCGCATCTT GGCCTTATTTTCAAGGAAAAGAGAGTGAAC GCAACTGGTACTGAAAAAGCAACACTGT	Common wheat lines/varieties CS, DS7E(7B), WGC002, PI 277012, Millennium, Wesley, Anton,Mace, Sumai 3, Wangshuibai, and Frontana; Durum line 2021MD1026	Cai et al. (2024)
13	*Fhb‐7E*	BE405003	PCR‐based, EST	Forward Reverse	GCCTCTAATGCAAGCTCTTTGA CTTGTGCATCCACCATAGATGA	Identifies alleles on 7E (*Th. elongatum*), 7A, 7B, 7D	Ceoloni et al. (2017); Kuzmanović et al. (2019)
14	*Fhb‐7el_2_*	CFA2240	SSR	Forward Reverse	TGCAGCATGCATTTTAGCTT TGCCGCACTTATTTGTTCAC	Identifies alleles on 7el_2_ (*Th. ponticum*), 7A, 7D	Ceoloni et al. (2017); Forte et al. (2014)
15	*Qfhb.nmbu.7A.2*	BS00098483_51	KASP	Forward 1 Forward 2 Reverse primer	TCAGATAAGCAGCAGGGACAT TCAGATAAGCAGCAGGGACAG GAAAGGGAATTATACGGTCCAGA	NMBU (300 European and exotic) spring wheat panel and the validation panel consisting of 358 new breeding lines	Nannuru et al. (2022)
16	*Qfhb.nmbu.7A.2*	AX‐95248570	KASP	Forward 1 Forward 2 Reverse primer	TGGGACTGGATGTGGTGAG TGGGACTGGATGTGGTGAA GCAAAGCAATAGGGCTAGGA	NMBU (300 European and exotic) spring wheat panel and the validation panel consisting of 358 new breeding lines	Nannuru et al. (2022)
17	*Qfhb.nmbu.7A.2*	Kukri_c57593_79	KASP	Forward 1 Forward 2 Reverse primer	CCACAGTAGGCTAAATGGACA CCACAGTAGGCTAAATGGACG ATCGTACACGCTCACTGCTG	NMBU (300 European and exotic) spring wheat panel and the validation panel consisting of 358 new breeding lines	Nannuru et al. (2022)

Abbreviations: EST, expressed sequence tag; GST, glutathione‐S‐transferase; KASP, kompetitive allele specific PCR; PACE, PCR Allele Competitive Extension; SNP, single nucleotide polymorphism; SSR, simple sequence repeat; STS, sequence‐tagged site.

### 7.3 Other approaches in resistance breeding

While marker‐assisted selection primarily uses a limited number of molecular markers tagging major QTL, genomic selection (GS) emerges as a promising approach to enhance FHB resistance in wheat. This method emphasizes the inclusion of many minor QTL, reducing both phenotyping efforts and costs (Arruda et al., 2016; Buerstmayr et al., 2020; Mir et al., 2023; J. Zhang, Gill, et al., 2022). GS also enhances the accuracy and intensity of selection within a constrained budget and timeframe, thereby maximizing the efficiency and effectiveness of the breeding programs. Specific studies have explored the potential of GS for FHB in bread wheat (e.g., Arruda et al., 2016; Garcia‐Abadillo et al., 2023; Haile et al., 2020; Rutkoski et al., 2012; Verges et al., 2021), and a few studies have focused on its use in durum wheat germplasm. Steiner, Michel, et al. (2019) conducted a comparative analysis of marker‐assisted, genomic, and phenotypic selection alternatives using a global collection of 228 durum wheat cultivars. Although phenotypic selection demonstrated the highest prediction ability, the outcomes from all genomic‐based selection methods, including marker‐assisted selection, were highly positive. These methods proved to be cost‐effective, particularly when major QTL were incorporated as fixed effects in the models. This approach increased prediction accuracy, a result consistent with findings in other bread wheat studies (Arruda et al., 2016; Boyles et al., 2024). The inclusion of secondary phenotypic traits in the model also enhances prediction accuracy. Gaire et al. (2022) demonstrated that including the percentage of *Fusarium* damaged kernels as a trait in a multi‐trait genomic model significantly increased the predictive abilities for DON accumulation in both validation and training sets in soft red winter wheat breeding diversity panels. J. Zhang, Gill, et al. (2022) observed similar improvements in a study involving 476 elite and advanced breeding lines from the South Dakota State University hard winter wheat breeding program. By incorporating the FHB disease index, the percentage of *Fusarium* damaged kernels, and days to heading as covariates in a multi‐trait model, the prediction accuracy for DON increased by 20% in a 1‐year trial.

Encouraging results have been reported regarding the use of morpho‐phenological traits to refine genomic predictions in durum wheat (Moreno‐Amores, Michel, Löschenberger, et al., 2020; Moreno‐Amores, Michel, Miedaner, et al., 2020). In a first study, significant enhancement in prediction accuracy was achieved by incorporating both flowering time and climatic data into the model, employing a multi‐trait model guided by anther retention, a morphologically neutral trait that shares a common genetic basis with FHB resistance (Moreno‐Amores, Michel, Löschenberger, et al., 2020). A subsequent study involving a diverse panel of 178 durum wheat lines evaluated across five environments demonstrated improved results when heading date and plant height were included as covariates. Specifically, treating heading date as fixed effect in the genomic prediction model yielded superior outcomes (Moreno‐Amores, Michel, Miedaner, et al., 2020).

To take full advantage of known genes within the *Fhb7* locus and likely associated disease‐resistance factors, chromosome engineering is an appealing option. Selection for single putatively responsible genes has already proven to be insufficient in capturing the desired resistance phenotype of the resistance donor, as seen with the *Thinopyrum* spp. *Fhb7 GST* candidate gene (X. Guo et al., 2022) and the *Fhb1* locus (Lagudah & Krattinger, 2019; G. Q. Li et al., 2019; Z. Ma et al., 2024; Su et al., 2019). Some seemingly contradictory results concerning these FHB QTL might be partially reconciled by interpreting these loci as complex loci or operon‐like clusters, that is, functionally related gene clusters assembled in close physical proximity, as reported for many eukaryotic organisms including plants (Boycheva et al., 2014). Notably, numerous clusters of *F. graminearum*‐responsive genes (FRGs), containing a variety of functionally related and largely co‐expressed defense genes, have been identified in the bread wheat genome (Perochon et al., 2021). Many of these clusters are physically close to and/or within known FHB QTL and contain both paralogous and non‐homologous genes implicated in plant stress and disease responses (including GSTs, UGTs, receptor‐like kinases, MAP kinases, nucleotide‐binding leucine‐rich repeats, and Cytochrome P450s; Perochon & Doohan, 2024). In line with the gene cluster theory, the *Fhb7* locus could encompass the horizontally acquired *GST* gene that expressed its contribution to FHB resistance when coupled with a favorable genetic background including functionally related genes (e.g., X. Guo et al., 2021; Konkin et al., 2022; H. Wang et al., 2020) that might be present in the distal *Thinopyrum* 7EL or 7el_2_L arm portions of the specific introgression lines. The same outcome would not be realized when only the *GST* gene is transgenically introduced (X. Guo et al., 2021, 2022). Co‐inheritance of beneficial genes/alleles within the *Fusarium*‐responsive cluster would be automatically facilitated by chromosomally engineered alien introgressions, as these segments are inherited as single Mendelian units. The genetic gain can be even further enhanced by nesting the *Fusarium*‐responsive cluster into a closely related alien segment with other useful traits. This approach has been applied to durum wheat recombinants equipped with a composite assembly of small segments originating from different *Thinopyrum* species or accessions (Forte et al., 2014; Kuzmanović et al., 2019).

## 8 PRIORITY ACTIONS FOR RESISTANCE IMPROVEMENT IN DURUM WHEAT

There is no single measure or silver bullet to achieve higher FHB resistance in durum wheat, but we have a clear picture about the components that are needed.

- A better genome‐wide coordinated pathogen and toxin surveillance should be carried out to monitor the evolution of *Fusarium* populations in durum wheat to be prepared for unforeseen changes in the pathogen population to more effectively select for FHB resistance. Notably, the Expert Working Group on Control of Wheat Pathogens of the Wheat Initiative aims to develop universal disease monitoring, diagnostic sampling, and detection strategies for FHB and other wheat diseases (https://www.wheatinitiative.org/ewg‐pathogens).
- A continued investment in reproducible phenotypic screening of large panels of breeding lines and any germplasm for FHB resistance traits. These activities should be continuously updated considering the new strains isolated through the abovementioned pathogen and toxin surveillance system.
- Improved lines have been generated in various programs around the globe, and we endorse exchange of material. As a first step, we advocate setting up a multi‐environment‐ring trial where all interested institutions share their improved lines, and a comprehensive side‐by‐side comparison of novel resistance sources will be performed for FHB response as well as phenological and morphological traits. Researchers and breeders would get access to diverse resistance sources, essential for combining these in regionally adapted cultivar candidates. The same panel should be genotyped with high‐density DNA fingerprints to clearly assess diversity. This unique germplasm and data set would greatly help in choosing the best genetic background for transferring resistant alleles identified in other sources.
- Progress on selection depends on choosing good parents. At least one parent should provide moderate‐to‐good FHB resistance if this trait is relevant for new cultivars. The list of already known potentially resistant durum lines (Table 3) is a strategic source of information for selecting resistance donors and markers linked to resistance QTL (Table 4). The initiative described in the previous point should bring this effort to an even more advanced level.
- It will remain challenging to develop regionally adapted, in most cases, semi‐dwarf and high‐quality cultivars. However, the selection of short stem lines with moderate‐to‐good resistance can be achieved, and increased anther extrusion can further support this approach. Continued recurrent selection has great potential to increase the resistance level of breeding populations and is therefore highly recommended (R. H. Wang et al., 2024).
- QTL‐based selection through marker‐assisted selection for major QTLs and GS for minor and unmapped QTLs is nowadays available and should be adopted as a modern breeding strategy aimed at leveraging at best the entire QTL iceberg for FHB resistance. Along this line, the QTL meta‐analysis summarized in Figure 1 presents the most complete and updated list of all QTLs governing reaction to FHB in common and durum wheat while providing relevant details on important pheno‐morphological traits also controlled, either pleiotropically an/or through linkage, by FHB resistance loci.
- In the longer term biotechnology tools, such a gene‐editing, for example, through knocking out susceptibility alleles, once these have been discovered and cloned, or adding resistance alleles through genetic engineering will provide additional options.

## AUTHOR CONTRIBUTIONS

**Ambra Viviani**: Data curation; formal analysis; writing—original draft. **Jemanesh Haile**: Conceptualization; data curation; formal analysis; writing—original draft. **Dilantha Fernando**: Data curation; formal analysis; writing—original draft. **Carla Ceoloni**: Writing—original draft. **Ljiljana Kuzmanovic**: Writing—original draft. **Dhondup Lhamo**: Writing—original draft. **Yong Gu**: Writing—original draft. **Steven Xu**: Writing—original draft. **Xiwen D. Cai**: Writing—original draft. **Hermann Buerstmayr**: Writing—original draft. **Elias Elias**: Writing—original draft. **Alessia Confortini**: Data curation; formal analysis; writing—original draft. **Matteo Bozzoli**: Data curation; formal analysis; writing—original draft. **Gurcharn Singh Brar**: Writing—original draft. **Yuefeng Ruan**: Writing—original draft. **Samia Berraies**: Writing—original draft. **Walid Hamada**: Writing—original draft. **Safa Oufensou**: Writing—original draft. **Malini Jayawardana**: Data curation; formal analysis; writing—original draft. **Sean Walkowiak**: Writing—original draft. **Salim Bourras**: Writing—original draft. **Monika Dayarathne**: Data curation; formal analysis; writing—original draft. **Julio Isidro Isidro**: Writing—original draft. **Fiona Doohan**: Writing—original draft. **Agata Gadaleta**: Conceptualization; writing—original draft. **Ilaria Marcotuli**: Writing—original draft. **Xinyao He**: Writing—original draft. **Pawan Kumar Singh**: Writing—original draft. **Susanne Dreisigacker**: Writing—original draft. **Karim Ammar**: Conceptualization; writing—original draft. **Valentyna Klymiuk**: Writing—original draft. **Curtis Pozniak**: Writing—original draft. **Roberto Tuberosa**: Writing—original draft; writing—review and editing. **Marco Maccaferri**: Data curation; formal analysis; writing—original draft; writing—review and editing. **Barbara Steiner**: Writing—original draft; writing—review and editing. **Anna Maria Mastrangelo**: Conceptualization; writing—original draft; writing—review and editing. **Luigi Cattivelli**: Conceptualization; writing—original draft; writing—review and editing.

## CONFLICT OF INTEREST STATEMENT

The authors declare no conflicts of interest.

## Supporting information




**Supplementary Data_S1**. Heritability of FHB traits in durum and bread wheat.

**Supplementary Data_S2**. Examples of DON‐responsive wheat genes/proteins existing or introduced via breeding into hexaploid wheat and proven to enhance either its’ DON tolerance and/or resistance to FHB.

**Supplementary Data_S3**. Projection of durum and bread wheat major and minor QTL and genes related to FHB response.

**Supplementary Data_S4**. QTL projection and identification of putative unique QTL‐clusters and singletones for FHB response detected in durum and bread wheat projected on Svevo RefSeq v1.0 genome.

## Data Availability

The authors declare that the data supporting the findings of this study are available within the article and its supplementary materials.
