# Supplementary material for: Priority actions for Fusarium head blight resistance in durum wheat: Insights from the wheat initiative
Source: Plant Genome. 2025 Jan 6;18(1):e20539. doi: 10.1002/tpg2.20539 (PMC11701714; doi:10.1002/tpg2.20539)
Supplement: Supplementary file 2 — Supplementary Data_S2. Examples of DON‐responsive wheat genes/proteins existing or introduced via breeding into hexaploid wheat and proven to enhance either its’ DON tolerance and/or resistance to FHB. [file TPG2-18-e20539-s004.docx]

**Supplementary DATA S2.** Examples of DON-responsive wheat genes/proteins existing or introduced via breeding into hexaploid wheat and proven to enhance either its DON tolerance and/or resistance to *Fusarium*^1^.

| Gene | Impact of DON and *Fusarium* on gene or protein^2^ | Experimental evidence for effect on DON tolerance and/or FHB resistance | Reference |
| --- | --- | --- | --- |
| Wheat UDP-glycosyltransferase *TaUGT6* | DON and *Fusarium* induced gene transcription in wheat spikes (higher in FHB resistant cv. Sumai-3 versus susceptible cv. Annong 8455) | Overexpression of chromosome 5B variant in wheat heads enhanced FHB resistance and decreased DON content | (He et al., 2020) |
| *Thinopyrum-derived* glutathione-S-transferase (within QTL *Fhb7*) | DON and *Fusarium* induced gene transcription in glumes of the wheat 7E2/7D substitution line | Translocation of a short encoding chromosome 7E fragment on wheat 7D resulted in wheat lines with broad resistance to FHB. Expression in yeast conferred it with DON tolerance. | (Wang et al., 2020) |
| Wheat cytochrome P450 *TaCYP72A* | DON and DON-dependent *Fusarium* induced gene transcription in spikelets of the FHB resistant, Sumai-3 derived, wheat cv. CM92036 | Virus-induced silencing (VIGS) concurrently targeting the chromosome 3A, 3B and 3D variants reduced DON tolerance in wheat heads | (Gunupuru et al., 2018) |
| Wheat multidrug transporter *TaABCC3* | DON induced gene transcription in wheat spikelets (higher in FHB resistant wheat cv. CM92036 than in the susceptible cv. Remus) | VIGS concurrently targeting variants on chromosome 3A and 3D reduced DON tolerance in spikelets | (Walter et al., 2015) |
| Wheat multidrug transporter *TaPDR7* | DON and *Fusarium* induced gene transcription in spikes of the FHB resistant wheat cv. Ning 7840 | VIGS concurrently targeting variants on chromosome 6A, 6B and 6D promoted growth of *F. graminearum* on wheat leaves | (Wang et al., 2016) |
| Wheat stress regulator *TaSnRK1α* | DON increased the protein kinase activity and the level of active (phosphorylated) TaSnRK1α in spikelets of the FHB resistant wheat cv. CM92036 | VIGS concurrently targeting chromosome 1 and 2 A, B and D variants reduced DON tolerance in spikelets. TaSnRK1α RNAi and gene overexpression respectivelt reduced and enhanced FHB disease resistance. | (Jiang et al., 2020; Perochon et al., 2019b) |
| Wheat NAC-like transcription factor *TaNACL-D1* | DON induced gene transcription in spikelets of the FHB resistant wheat cv. CM92036 | Gene overexpression enhanced FHB resistance in wheat heads | (Perochon et al., 2019a) |
| Wheat taxonomically restricted novel gene *TaFROG* | DON and DON-dependent *Fusarium* induced gene transcription in spikelets of the FHB resistant wheat cv. CM92036 | VIGS of chromosome 4A, 4B and 4D variants reduced DON tolerance of spikelets; overexpression of the 4A variant enhanced FHB resistance | (Jiang et al., 2020; Perochon et al., 2019b) |

^1^Many other genes have been associated with DON tolerance and FHB resistance, but the experimental evidence for the direct effect on these traits is not yet available. Additionally, an Arabidopsis lipid transfer protein, discovered as part of a screen of activation tagged mutants for those with enhanced trichothecene resistance, was shown to enhance FHB resistance and suppress DON accumulation when expressed in wheat (McLaughlin et al., 2021).

^2^DON-dependent ‘*Fusarium* induced’ means expression was detected in response to wild type *F. graminearum* but was minimal or absent in response to its DON-minus mutant derivative. ‘*Fusarium* induced’ means it was induced by the pathogen, but the importance of DON production by the fungus for this induction is unknown.

**References**

Gunupuru, L. R., Arunachalam, C., Malla, K. B., Kahla, A., Perochon, A., Jia, J., Thapa, G., & Doohan, F. M. (2018). A wheat cytochrome P450 enhances both resistance to deoxynivalenol and grain yield. *Plos One, 13(10),* e0204992. https://doi.org/10.1371/journal.pone.0204992

He, Y., Wu, L., Jiang, P., Zhang, X., & Ma, H. (2020). *TaUGT6*, a novel UDP-glycosyltransferase gene enhances the resistance to FHB and DON accumulation in wheat. *Frontiers in Plant Science, 11,* 574775. https://doi.org/10.3389/fpls.2020.574775

Jiang, C., Hei, R., Yang, Y., Zhang, S., Wang, Q., Wang, W., Zhang, Q., Yan, M., Zhu, G., Huang, P., Liu, H., & Xu, J.-R. (2020). An orphan protein of *Fusarium graminearum* modulates host immunity by mediating proteasomal degradation of TaSnRK1α. *Nature Communications, 11(1),* 4382. https://doi.org/10.1038/s41467-020-18240-y

Perochon, A., Kahla, A., Vranić, M., Jia, J., Malla, K. B., Craze, M., Wallington, E., & Doohan, F. M. (2019a). A wheat NAC interacts with an orphan protein and enhances resistance to Fusarium head blight disease. *Plant Biotechnology Journal, 17(10),* 1892-1904. https://doi.org/10.1111/pbi.13105

Perochon, A., Váry, Z., Malla, K. B., Halford, N. G., Paul, M. J., & Doohan, F. M. (2019b). The wheat SnRK1α family and its contribution to Fusarium toxin tolerance. *Plant Science, 288,* 110217. https://doi.org/10.1016/j.plantsci.2019.110217

Walter, S., Kahla, A., Arunachalam, C., Perochon, A., Khan, M. R., Scofield, S. R., & Doohan, F. M. (2015). A wheat ABC transporter contributes to both grain formation and mycotoxin tolerance. *Journal of experimental botany*, *66*(9), 2583-2593. https://doi.org/10.1093/jxb/erv048

Wang, G. P., Hou, W. Q., Zhang, L., Wu, H. Y., Zhao, L. F., Du, X. Y., Xin, M., Li, A. F., Wang, H. W., & Kong, L. R. (2016). Functional analysis of a wheat pleiotropic drug resistance gene involved in Fusarium head blight resistance. *Journal of integrative agriculture, 15(10),* 2215-2227. https://doi.org/10.1016/S2095-3119(16)61362-X

Wang, H., Sun, S., Ge, W., Zhao, L., Hou, B., Wang, K., Lyu, Z., Chen, L., Xu, S., Guo, J., Li, M., Su, P., Li, X., Wang, G., Bo, C., Fang, X., Zhuang, W., Cheng, X., Wu, J., Dong, L., Chen, W., Li, W., Xiao, G., Zhao, J., Hao, Y., Xu, Y., Gao, Y., Liu, W., Liu, Y., Yin, H., Li, J., Li, X., Zhao, Y., Wang, X., Ni, F., Ma, X., Li, A., Xu, S. S., Bai, G., Nevo, E., Gao, C., Ohm, H., & Kong, L. (2020). Horizontal gene transfer of *Fhb7* from fungus underlies *Fusarium* head blight resistance in wheat. *Science, 368,* eaba5435. <https://doi.org/10.1126/science.aba5435>
